# Low-Dimensional Zeotypes Templated by Stacked Cyclic Benzimidazolium Revealed by Electron Crystallography

**DOI:** 10.1021/jacs.5c22569

**Published:** 2026-02-05

**Authors:** Evgeniia Ikonnikova, Jung Cho, Xiaodong Zou, Andre Sutrisno, Allen W. Burton, Trong Pham, Tom Willhammar

**Affiliations:** a Department of Chemistry, 7675 Stockholm University, Stockholm SE-106 91, Sweden; b ExxonMobil Technology & Engineering Company, 1545 Route 22 East, Annandale, New Jersey 08801, United States

## Abstract

The structural diversity of zeolites depends strongly on the use of organic structure-directing agents (OSDAs) that guide their formation. Low-dimensional zeolitic materials, such as layered or chain-like phases, can serve as key intermediates in topotactic condensation pathways, yet the mechanisms governing their formation and transformation remain poorly understood. Here, we report three low-dimensional zeolitic materials, EMM-75P, EM-L01, and EM-L02, synthesized using benzimidazolium cations as OSDAs. Their structures were determined by three-dimensional electron diffraction (3D ED), including the atomic structure of the OSDAs, revealing their confinement within the framework to shed light on their structure-directing role. The bulky benzimidazolium OSDAs prevent the formation of materials with three-dimensional framework structures and instead direct the formation of low-dimensional zeotypes. Upon calcination, the two-dimensional layered aluminosilicate zeotype EMM-75P undergoes topotactic condensation to form a three-dimensional zeolite, EMM-75, with a previously unreported zeolite framework topology. Aluminosilicate EM-L01 is a 2D analogue of **STF**/**SFF** zeolite frameworks and partially condensed to an **STF**-topology upon calcination, whereas EM-L02, a 1D silicate composed of double 6-ring chains, packed analogous to the **CHA** zeolite framework, collapses during the thermal treatment. The detailed structural characterization of these three materials provides insights into the mechanism of topotactic condensation and demonstrates how such pathways can lead to new zeolite materials.

## Graphical abstract

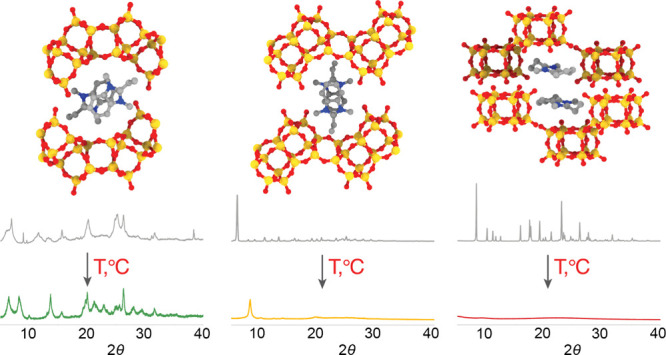

## Introduction

Zeolites are generally defined as crystalline microporous aluminosilicates with three-dimensional structures built from corner-sharing tetrahedrally coordinated atoms. Zeolites contain one-, two-, or three-dimensional channel systems that may be interconnected. According to the Structural Commission of the International Zeolites Association,^1^ 264 different framework structures have been accepted so far. Two-dimensional (2D) layered zeolitic materials possess a structure extending only in two dimensions, with a framework interrupted in the third dimension. They may condense to a 3D periodic zeolite structure upon calcination by topotactic silanol condensation; such 2D zeolitic materials are referred to as 2D layered zeolitic precursors. A limited number of such 2D layered zeotype materials have emerged, among them EU-19 (**CAS**),^2^ PREFER (**FER**),^3^ MCM-22P (**MWW**),^4^ Nu-6(1) (**NSI**),^5,6^ EMM-9 (**SFO**),^7^ RUB-15 (**SOD**),^8^ CIT-8P (**HEU**),^9^ EU-12 (**ETL**),^10^ and HPM-3 (**JSN**).^11^ PST-9 is a notable recent addition where a layered phase transforms hydrothermally into the small pore zeolite EU-12 (**ETL**) through a phase transformation. Another interesting example is the topotactic condensation of a layered aluminosilicate precursor CIT-8P to CIT-8 (**HEU**) by synthesis using a diquaternary organic structure-directing agent. 1D zeotype materials are rare, with one recent example being the silica chain ZEO-2, which can be converted into a 3D zeolite upon calcination.^12^ Zeotypes are typically formed using hydrothermal synthesis where the choice of organic structure-directing agent (OSDA) is one of the most important parameters to guide the formation of a desired material. An alternative approach to obtain zeolites using 2D zeolitic materials is the Assembly, Disassembly, Organization, and Reassembly (ADOR) method, where 2D zeolitic precursors are obtained from 3D germanosilicate zeolites followed by a guided assembly to form new zeolite materials.^13,14^ With an increased understanding of the role of the OSDA in the formation of low-dimensional zeotype materials, new materials, currently unfeasible by direct hydrothermal synthesis, can be obtained.^15^

Several databases compile information about zeolite synthesis conditions and enable prediction of zeolite synthesis and phase competition using large-scale simulations and machine learning (ML).^16–20^ In contrast, when it comes to low-dimensional zeolitic materials, databases are still at an early stage, given that these materials have been less explored.^21,22^ An intriguing aspect of zeolitic materials is the opportunities for postmodification via pillaring,^23^ delamination,^24,25^ intercalation, etc., to synthesize derivative structures. Often, the bottleneck in the growth of zeolites via conversion from low-dimensional materials to a 3D zeolite is the condensation process, where the OSDA degradation occurs. By learning more about the structure-directing mechanisms of the OSDA in the formation of low-dimensional zeotypes and the subsequent condensation to form a 3D zeolite, the trial-and-error approach in the synthesis of novel zeolites from low-dimensional precursors can be minimized. Therefore, improved comprehension could pave the way toward a more systematic approach driven by ML for designing novel low-dimensional zeotypes.

In general, the structure elucidation of zeotype materials and specifically low-dimensional zeotypes poses challenges due to their small crystal size in combination with large unit cell volume. This prevents the use of single-crystal X-ray diffraction (SCXRD) and results in peak broadening and significant peak overlap in powder X-ray diffraction (PXRD) patterns. The three-dimensional electron diffraction (3D ED) method has been shown to be highly advantageous for determining complex structures of materials with a submicrometer crystal size.^26–28^ The electron probe interacts much more strongly with matter compared to X-rays, enabling the acquisition of single-crystal data from small crystals and thereby enabling accurate crystal structure determination, including the location of the organic structure-directing agent.^29,30^ Understanding the interactions between the framework and OSDA provides valuable insight into the structure-directing role of the organic molecule in the formation of a framework, as well as possibly the location of the active site within the framework.^31^

Herein, we present the structure determination of three new low-dimensional zeotype materials, the 2D layered phases EMM-75P and EM-L01 synthesized using 2-ethyl-1,3-dimethylbenzimidazolium as OSDA molecule, and the 1D silicate, EM-L02, synthesized with a similar hydrogenated OSDA, 2-ethyl-1,3-dimethyl-4,5,6,7-tetrahydrobenzimidazol-3-ium. We demonstrate the formation of a new zeolite framework, EMM-75, by topotactic condensation of the layered EMM-75P. Moreover, the atomic structures of the OSDAs within the crystals were revealed using 3D ED data for all three as-made zeotype materials and their interaction with the framework was described. The structural changes upon calcination were investigated via in situ PXRD, thermogravimetric analysis (TGA), differential scanning calorimetry (DSC), nuclear magnetic resonance (NMR), and scanning transmission electron microscopy (STEM) images. Together, these methods provide important insights into the role of the OSDA in the formation of low-dimensional zeolitic materials and their structural transformations upon removal of the OSDA to form a 3D zeolite material.

## Results and Discussion

EMM-75P and layered EM-L01 were synthesized using the same diquaternary OSDA, 2-ethyl-1,3-dimethylbenzimidazolium (OSDA1, Figure 1a), and EM-L02 was prepared using the partially saturated form 2-ethyl-1,3-dimethyl-4,5,6,7-tetrahydrobenzimidazol-3-ium (OSDA2, Figure 1b). The structural integrity and spatial confinement of OSDAs occluded within the structure of the zeotype materials were probed by ^1^H/^13^C cross-polarization magic-angle spinning (CPMAS) NMR (see Figures S1a, S2a, and S3a). The gel composition in the synthesis of EMM-75P was Si/Al = 15, OSDA1­(OH)/Si = 0.5, H_2_O/Si = 10, HF/Si = 0.5. The synthesis conditions for EM-L01 were identical to EMM-75P, except for Si/Al = 30. EM-L02 resulted from a synthesis gel with composition H_2_O/Si = 4, OSDA2­(OH)/Si = 0.5, HF/Si = 0.5. HF was used to control the pH during synthesis in the presence of hydroxide, in order to ensure OSDA stability. The fluorine environment in EMM-75P, EM-L01, and EM-L02 was studied using ^19^F MAS NMR and revealed both sharp and broad resonance, which were attributed to fluoride species interacting with various framework and extra-framework components (Figures S1b, S2b, and S3b).

**Figure 1:**
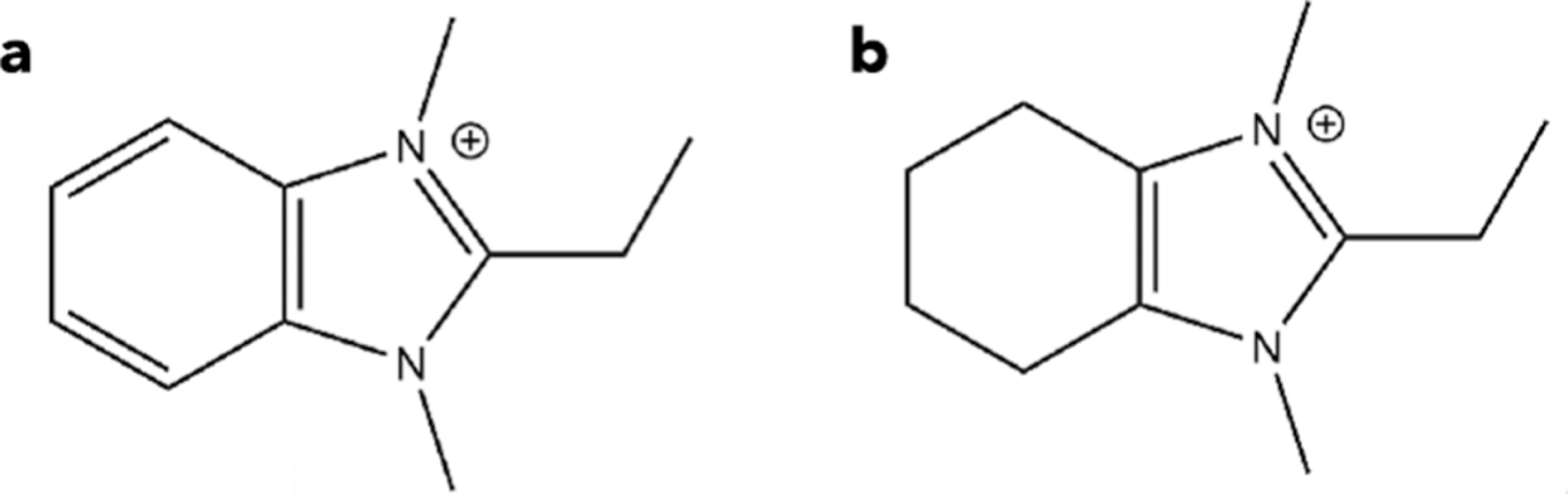
Organic structure-directing agents used in the synthesis. (a) 2-Ethyl-1,3-dimethylbenzimidazolium (denoted OSDA1) used to form EMM-75P and EM-L01 and (b) 2-ethyl-1,3-dimethyl-4,5,6,7-tetrahydrobenzimidazol-3-ium (denoted OSDA2) used to form EM-L02.

All three phases in their as-synthesized forms exhibit PXRD patterns indicative of crystalline phases (see Figures S4a,c,e). The ^27^Al MAS NMR spectra (Figure S5a) reveal that EMM-75P predominantly contains tetrahedrally coordinated framework aluminum species (Al^IV^), accounting for approximately 90% of the total aluminum signal with a minor broad resonance near 0 ppm attributed to hexacoordinated extra-framework aluminum species (Al^VI^). The ^29^Si NMR spectrum (Figure S6a) of EMM-75P shows peaks consistent with tetrahedrally coordinated Si species, Q^4^(0Al) and Q^4^(1Al) as well as silanol groups (≡Si–OH, Q^3^). Based on peak deconvolution, NMR suggests a silanol content of 15.4% in close proximity to the theoretical value (16.7%).

In contrast, the ^27^Al MAS NMR spectrum of EM-L01 exhibits a strong signal corresponding to tetrahedral framework Al^IV^, with no detectable resonance at 0 ppm, indicating the absence of significant extra-framework Al species (Figure S5b). A sharp, low-intensity peak (∼2%) at ∼20 ppm is assigned to hexacoordinated aluminum (Al^VI^), possibly originating from trace Al_2_O_3_ impurities.^32^ The ^29^Si NMR spectrum shows resonances indicative of fully condensed Q^4^ Si­(0Al) environments, with a shoulder indicative of Q^3^ species (see Figure S7a).

The ^29^Si MAS NMR spectrum of EM-L02 (Figure S8) indicates a significant degree of framework connectivity of fully condensed silica species (Q^4^, Si­(0Al)) with notable signals attributed to Q^3^ species.

Scanning electron microscopy (SEM) images reveal plate-like crystal morphologies of EMM-75P and EM-L01 and a rod-like shape of the EM-L02 phase (see Figure 2). Upon calcination in air, the PXRD data indicates the transformation of EMM-75P to a new crystalline phase (Figure S4b), whereas the PXRD patterns of EM-L01 and EM-L02 only show partial (Figure S 4d) and no crystallinity (Figure S 4f), respectively. The ^29^Si NMR spectrum of calcined EMM-75 shows no distinct peak corresponding to Q^3^ species.

**Figure 2:**
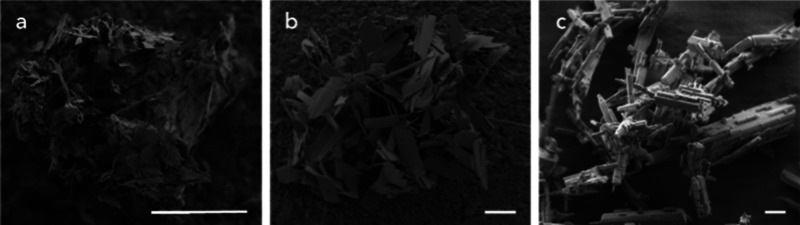
SEM images show the crystal morphology of (a) EMM-75P, (b) EM-L01, and (c) EM-L02 (scale bars 5 μm).

### Structure Determination of Zeotype Materials

3D ED data for EMM-75P were collected at room temperature. Three 3D ED data sets of EMM-75P were merged for structure solution and refinement, in order to improve completeness (Table S1). The 3D reciprocal lattice was reconstructed based on the 3D ED frames (Figure S9) and indexed using a monoclinic unit cell, with averaged unit cell parameters of *a* = 7.33(1) Å, *b* = 17.73(1) Å, *c* = 25.17(1) Å, and β = 92.54(3)°. Systematic absences were consistent with the space group *P*2_1_/*n* (no. 14). All atoms of the structure, including T and O atoms of the zeolitic layer as well as the 2-ethyl-1,3-dimethylbenzimidazolium molecule (Figure 3a,d), were found ab initio from the structure solution. During least-squares refinement, constraints were used to optimize the bond lengths and angles of some of the framework atoms as well as the OSDA. The refinement was stable and converged to an *R*_1_ value of 18.5%.

**Figure 3:**
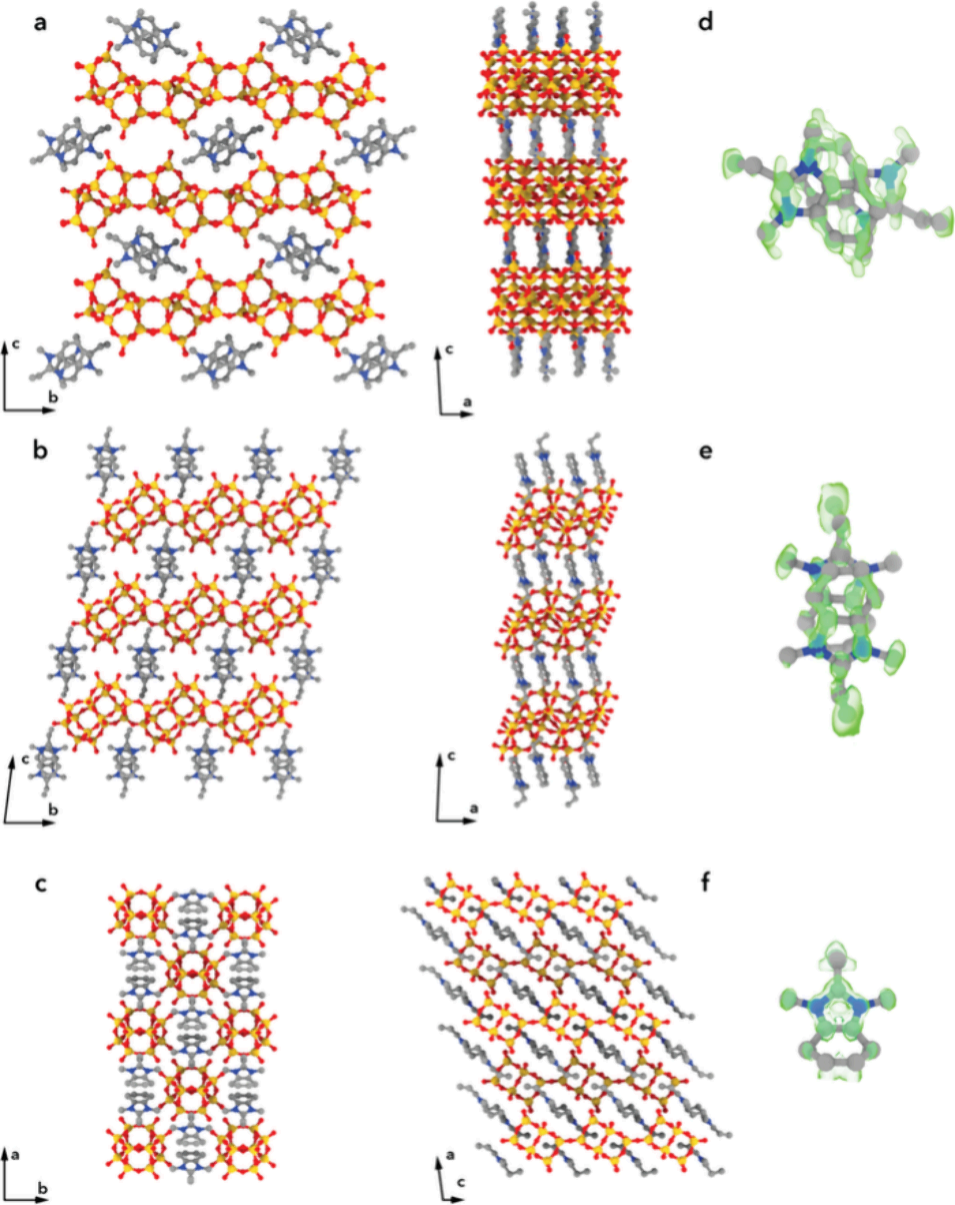
Refined structures of (a) EMM-75P, (b) EM-L01, and (c) EM-L02 viewed along two perpendicular directions. (d–f) The difference electrostatic potential map, shown in green, revealed the atomic structure of the organic structure-directing agents intact between the inorganic units for (d) EMM-75P, (e) EM-L01, and (f) EM-L02. T atoms are shown in yellow, oxygen in red, carbon in gray, and nitrogen in blue.

Structure solution of EM-L01 was performed from a single 3D ED data set collected at room temperature (Figure S10a). However, in order to achieve a higher completeness, six data sets were merged for structure refinement (Table S2). The structure was solved in the triclinic space group *P*-1 (no. 2), and the average unit cell parameters from 3D ED data were *a* = 7.41(2) Å, *b* = 11.43(2) Å, *c* = 14.73(6) Å, α = 79.38(9)°, β = 87.65(25)°, and γ = 83.29(6)° (Table S2). All non-hydrogen atoms were obtained directly from the structure solution, including those of the OSDA, indicating a highly ordered character of the organic molecule between the layers. The structure of EM-L01 contains the same layers shared in the **STF** and **SFF** zeolite frameworks;^33^ to our knowledge, this is the first time they are reported as a layered zeotype.

EMM-75P and EM-L01 are both synthesized using the same OSDA. In both structures, the OSDA molecules are packed face-to-face with π–π stacking at an intermolecular distance of 3.5–3.7 Å to form one-dimensional stacks along the *a*-axis (see Figure 4a,b). For EMM-75P, the OSDA stack is oriented with its longer cross-section dimension parallel to the zeolitic layers (see Figure 3a), whereas for EM-L01, the OSDA-stack is oriented with the longer cross-section dimension perpendicular to the layers (Figure 3b). This results in a larger interlayer spacing and distance between terminating hydroxyl groups of the zeolitic layers of 3.8 Å in contrast to 2.7 Å in EMM-75P. In each of the 2D layered structures, the 3D ED refinements show well-defined OSDA positions, indicating low mobility of the molecules between the layers due to their rigidity and π–π interactions. The use of a similar OSDA, 2-ethyl-1,3-dimethylimidazolium, has been reported by Schmidt et al.^34^ to yield a pure-phase silicate **STF** zeolite, as well as a mixture of **STW**, **ITW**, and **MTW**. This suggests that the use of a bulkier OSDA, 2-ethyl-1,3-dimethylbenzimidazolium, in this work, with an additional benzene ring, promotes the formation of a layered phase. A bulkier OSDA imposes steric restrictions preventing the formation of the 3D zeolite, and the strong π–π stacking of the benzene ring also helps to stabilize the layered phase and promote channel formation.

**Figure 4:**
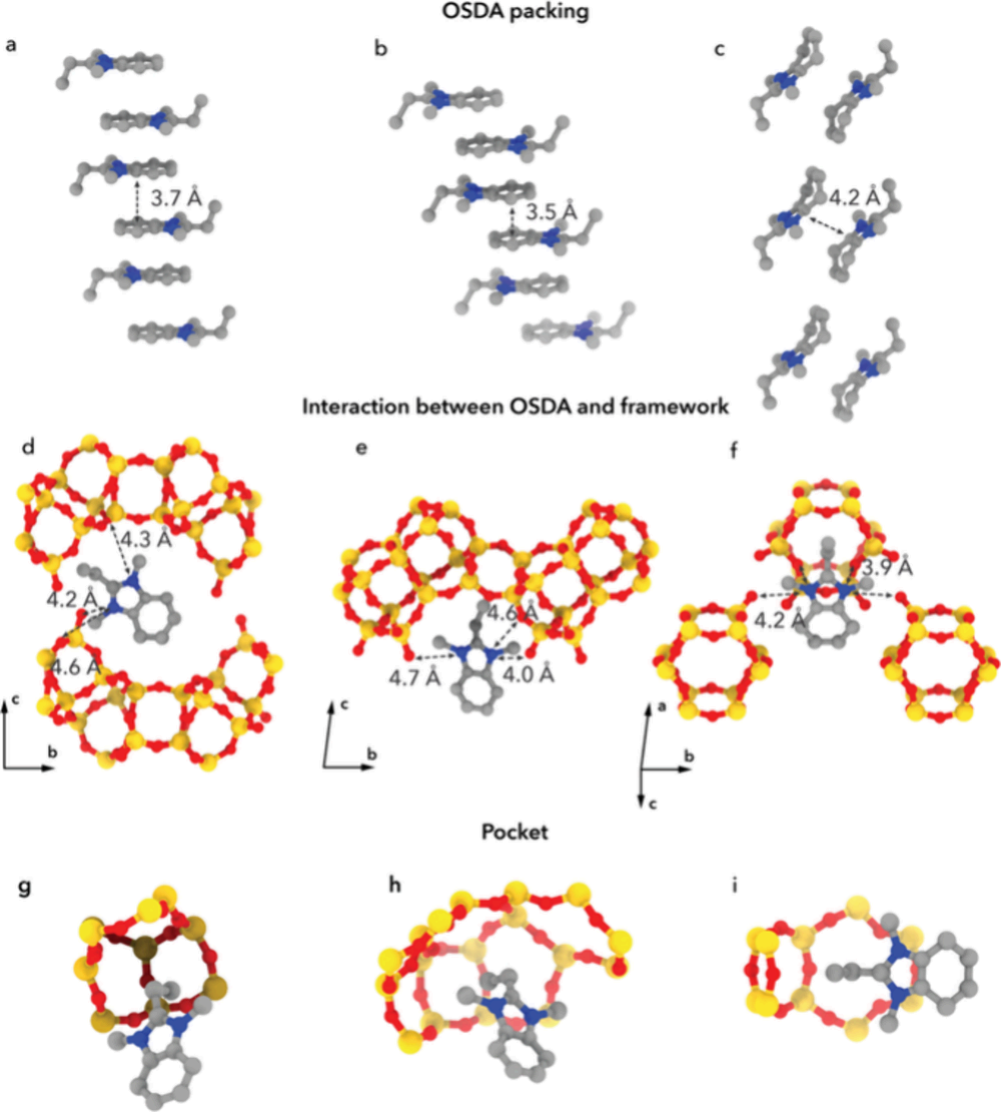
Packing of the OSDA molecules in the structures of (a) EMM-75P, (b) EM-L01, and (c) EM-L02, respectively. (d–f) Location of the OSDA molecules in each of the materials, with the closest distances between nitrogen of the imidazolium and framework oxygen atoms for each of the materials indicated. (g–i) Steric effect of the OSDA inside the frameworks. T atoms are shown in yellow, oxygen in red, carbon in gray, and nitrogen in blue.

The structure determination of EM-L02 was performed using a single 3D ED data set collected at room temperature with a 119.3° rotation range (Figure S11 and Table S3). The structure was obtained in a monoclinic crystal system with space group *C*2/*m* (no. 12) and *a* = 17.09(1) Å, *b* = 14.51(1) Å, *c* = 9.25(1) Å, and β = 108.80(3)°. All atoms of the inorganic unit as well as the OSDA were obtained from the structure solution, including those of the flexible alkyl groups, further confirming the rigid position of the OSDA (see Figure 3c,f). Structure refinement was performed using constraints on the bond lengths of the cyclohexane and converged to *R*_1_ of 18.8%. The Si–O distance was close to ideal, with an average of 1.61 Å, and all bond lengths and angles of OSDA2 were reasonable. The structure of EM-L02 possesses a one-dimensional chain of double 6-rings (*d6r*) linked by oxygen atoms forming 4-rings (see Figure 3c). The chains are arranged at hydrogen bonding distances between terminal hydroxyl groups and are arranged similarly as in zeolite framework structure **CHA**. The space between chains is filled by OSDA2 molecules arranged in pairs, which are packed to form 1D rods extending along the *c*-axis. EM-L02 differs fundamentally from previously reported one-dimensional silicates by its construction from double six-ring units linked into extended parallel chains. The structure constitutes a 1D zeotype analogue of the CHA framework, offering a potential platform for postsynthetic assembly into higher-dimensional zeolitic architectures.

The Pawley fit of PXRD data using TOPAS Academic V5 is consistent with the space groups and unit cell parameters for all three low-dimensional zeotypes and zeolite EMM-75 (Figure S20 and Table S5). The Pawley fit results verified that EMM-75P, EM-L01, and EMM-75P are single-phase materials. Also, EM-L02 is the predominant phase in the material, with a minor content of zeolite Beta found.

The distances between terminal oxygen atoms of neighboring units in EMM-75P and EM-L02 are in the range of 2.6–2.7 Å, indicating hydrogen bonding, whereas for EM-L01, the terminal hydroxyl groups are at a distance of 3.8 Å, which is too far apart for hydrogen bonding. A closer look at the OSDA to inorganic unit distance can further explain the packing. For EMM-75P, each nitrogen atom of a single OSDA molecule is located at a distance of 4.2–4.6 Å from oxygen atoms of both adjacent zeolitic layers, further stabilizing the layered structure (see Figure 4d). In EM-L01, the nitrogen atoms of each OSDA1 molecule are just located in conjunction with one of the zeolitic layers at distances of 4.0–4.7 Å to the closest oxygen atom (Figure 4e). However, the π–π stacked OSDA1 molecules are alternatingly located closely to layers above and below to stabilize the layered structure. In EM-L02, each nitrogen of the OSDA2 molecule is interacting at a distance of 3.9–4.2 Å with the nearest oxygen atoms of two neighboring inorganic units to bring further stability to the structure, in addition to the hydrogen bonding between terminal hydroxyl groups (see Figure 4f). In EMM-75P, the ethyl group of the OSDA is oriented toward the terminal T-site, whereas in EM-L01, it is directed toward the center of a 4-ring; in EM-L02, it points toward one of the oxygen atoms of a 4-ring (Figure 4d–f). In all structures, the steric effect of the ethyl group creates a pocket within the structure of the layer (Figure 4g–i). The atomic-level understanding of the OSDA location underscores the importance of 3D ED not only in determining framework structures but also in revealing structural details, thereby deepening the understanding of low-dimensional zeotype chemistry.

### Structure Determination of Zeolite EMM-75

After calcination of EMM-75P, the PXRD pattern reveals a well-crystalline phase, EMM-75, with a distinct pattern (see Figure S4b). The ^29^Si NMR spectrum (Figure S6b) of EMM-75 is similar to the results for EMM-75P, however, with a reduced intensity around −100 ppm, indicating reduced content of Q^3^ silanol groups. Exact quantification is, however, challenging due to the limited resolution of the NMR spectrum, likely caused by the morphology of EMM-75 crystals and possibly incomplete condensation.

3D ED data of EMM-75 were obtained at room temperature, and structure determination was performed based on a single data set (Table S4). The crystal system of EMM-75 is orthorhombic with the space group *Pnnm* (no. 58) based on the reflection conditions 0*kl*: *k* + *l*, *h*0*l*: *h* + *l* (Figure S12), and the unit cell parameters are *a* = 7.36(10) Å, *b* = 17.77(32) Å, and *c* = 21.57(72) Å. The resulting 3D framework structure is fully four-connected with 6 T and 14 O atoms in the asymmetric unit. The final refinement converged to *R*_1_ of 19.3%.

### Structure Description of EMM-75P and EMM-75

The structure of EMM-75 is formed by topotactic condensation of EMM-75P along the *c*-axis to yield a 3D framework (see Figure 5). The structure of EMM-75 exhibits a 2D pore system with large 12- and small 8-ring channels along the *a*-axis interconnected by 8-ring windows along the *b*-axis. The 12-rings have a dimension of 8.1 × 6.2 Å, and the 8-rings along the *b*-axis are distorted with dimensions of 5.7 × 2.4 Å (after subtracting 2.7 Å corresponding to two oxygen radii). The pore structure with 12- and 8-ring channels running in parallel, interconnected by 8-ring windows, show similarities to industrially relevant zeolite topologies such as **MOR**.

**Figure 5:**
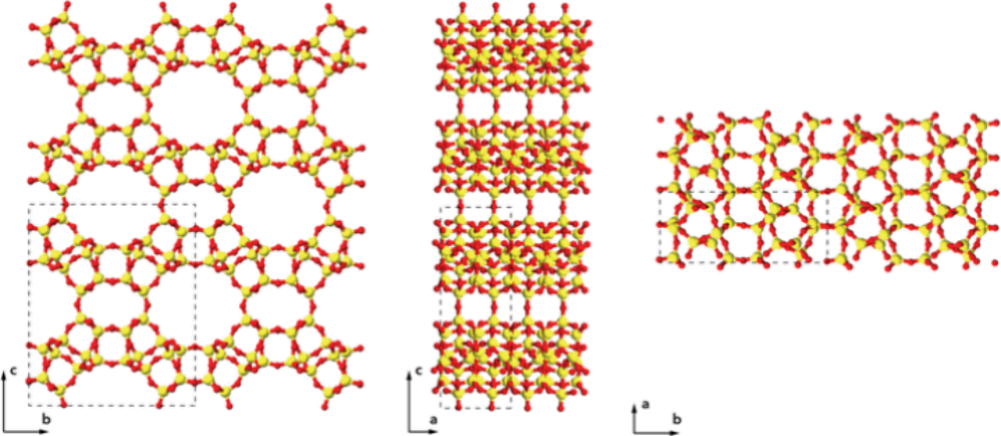
Refined structure of EMM-75 viewed along main directions. The 12- and 8-ring channels are running along the *a*-axis and are interconnected by distorted 8-ring windows along the *b*-axis. T atoms are shown in yellow and oxygen in red.

Since the structure of EMM-75 could not be described using existing composite building units in the IZA database, a tiling analysis was performed with ToposPro^35^ software. The framework structure of EMM-75 was found to be constructed of two tilings, *t-euo* and *t-kaj* (Figure S13a,b), which are also found in the **MON** ^36^ framework, but connected in a different arrangement to form a framework. Periodic nets can be constructed by first connecting *t-euo* tiles ([4.5^2^]) related by a two-fold screw-axis operation to obtain one-dimensional rods extending along the *a*-axis. These rods are then combined along the *b*-axis, related by an *a*-glide plane operation to form layers in the *ab*-plane, which construct the framework structures of EMM-75P and EMM-75 (Figure 6). This tiling fully describes the structures of both EMM-75P and EMM-75. The *t-euo* tile is also present in the layered MWW-type zeolitic materials.

**Figure 6:**
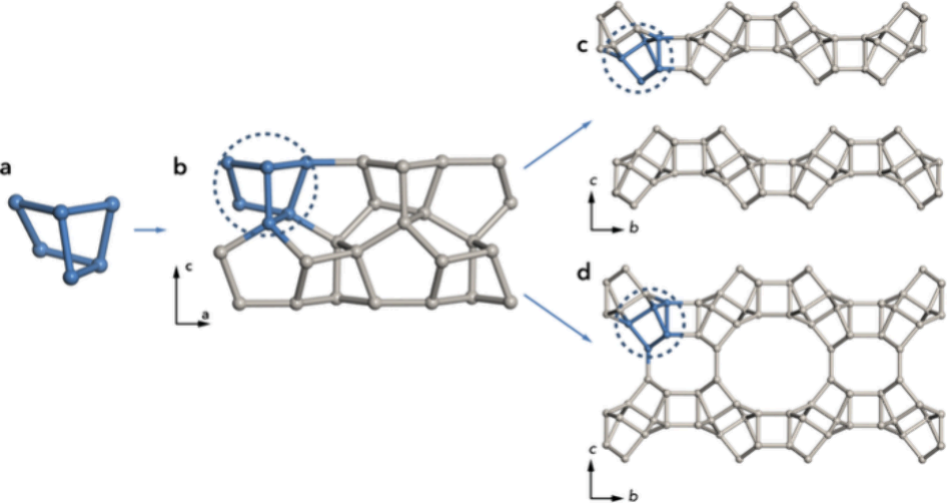
Frameworks of EMM-75P and EMM-75 are constructed by the (a) *t-euo* natural tiling, (b) combined into 1D rods extending along the *a*-axis, which are combined along the *b*-axis into the layers building up the structures of (c) EMM-75P and (d) EMM-75.

### Structure Transformation of Low-Dimensional Materials upon Calcination

In order to understand the structural transformation occurring during calcination, in situ PXRD, thermogravimetry, and differential scanning calorimetry were utilized. In situ PXRD measurement of EMM-75P showing transformation into EMM-75 was conducted in a temperature range from 100 °C to 560 °C in air with a heating rate of 3 °C/min (Figure 7). The PXRD patterns at the start and end points could be indexed using the structures of EMM-75P and EMM-75, respectively, as determined using 3D ED. A clear indication of topotactic condensation was observed as the decrease of the unit cell dimension along the *c*-axis, evidenced by the shift of 002 reflection from ∼7.1° (corresponding to a *d*-spacing of 12.5 Å) at 100 °C to ∼8.4° (10.5 Å) 2θ at 560 °C. The changes can be divided into several stages. First, before 350 °C, the 002 peak is getting sharper due to increasing crystal size, whereas the broad 011 peak is losing intensity, possibly due to water desorption. At the second stage, at 400 °C when OSDA starts to degrade, the 002 peak starts to shift toward higher angles as the interlayer distance is decreasing and the condensation of the silanol groups is progressing. The intensity of the 011 peak is now increasing as the OSDA molecules are removed from the interlayer space of the structure and the 002 and 011 peaks are getting sharper as the condensation results in larger ordered crystals.

**Figure 7:**
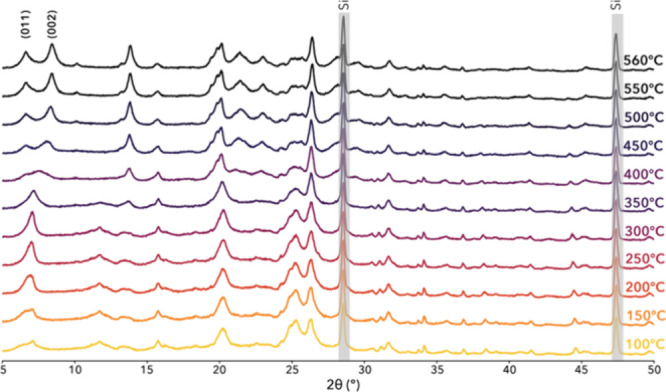
In situ PXRD investigation of EMM-75P with a step of 3 °C/min reveals the topotactic condensation to form EMM-75 with an onset temperature at 350 °C (λCuKα1 = 1.5406 Å). Si powder (peaks in gray) was used as an internal standard.

The TGA curve of the calcination of EMM-75P in air shows a slope characteristic of a layered material with OSDA molecules stabilized in the interlayer position (Figure S16). The weight loss below 200 °C can be attributed to water desorption, accounting for 9.8% of the total weight loss, while the weight loss from 300 °C can be assigned to the OSDA degradation and the water released from silanol condensation, giving a weight loss of 19.5% in agreement with the calculated weight loss of 21.1%. The OSDA content is also consistent with a CHN analysis showing an organic content of 22.3% compared to a theoretical value of 19.2%. Additionally, a single step in the DSC curve signifies the thermal decomposition of OSDA molecules, characterized by an exothermic process with a peak maximum at 487 °C.

Neither EM-L01 nor EM-L02 forms a highly crystalline three-dimensional framework material upon calcination. According to in situ PXRD data of EM-L01, the 001 peak starts to shift continuously toward higher 2θ angles after 250 °C, shifting from a *d*-spacing of 14.4 Å at 250 °C to 10.7 Å at 600 °C (see Figure S14). Beyond 500 °C, an increase in intensity and sharpening of the 001 peak was observed with the absence of long-range ordering beyond the interlayer stacking. The DSC reveals an exothermic process occurring from 270 °C (Figure S17), which can be associated with the onset of the degradation of the OSDA in good consistency with the in situ PXRD data. The TGA data for EM-L01 exhibits a total weight loss of 34.7% with 23.8% occurring from 300 °C, attributed to the decomposition of the OSDA and condensation of silanol groups (Figure S17). This observation is consistent with the calculated value of 28.7%. The OSDA content is 24.6% according to CHN analysis (26.0% theoretically). The 001 peak remains intense and sharp, indicating that the layer is preserved, although there is no ordered topotactic condensation. The ^29^Si NMR spectrum of EM-L01 shows a significant broadening of the signal upon calcination and a reduction in signal intensity around −100 ppm attributed to Q^3^ silanol species, indicating partial condensation and a less well-ordered material (see Figure S7b). Thus, the powder after TG analysis (denoted EM-L01-TG) was further studied by 3D ED and STEM images. 3D ED data of EM-L01-TG revealed pronounced diffuse streaks along the c*-axis, while the periodicities along a* and b* remained consistent with the as-made material (Figure S10b). A successful structure determination was nevertheless performed from several collected data sets where the streaks in reciprocal space were less pronounced. Indexing these data sets revealed unit cell parameters *a* = 14.14(24) Å, *b* = 18.44(11) Å, *c* = 7.47(33) Å, and β = 99.18(15)° and the space group of *C*2/*m* (no. 12). Structure solution yielded the framework structure of the **STF** framework. Likely, the larger interlayer distance of 3.8 Å between adjacent hydroxyl groups in EM-L01, in contrast to the 2.7 Å spacing between oxygens in the EMM-75P, prevents the full successful topotactic condensation to a 3D zeolite framework, which is consistent with earlier studies.^11,12,38^

In situ PXRD of EM-L02 shows a well-crystalline sample, which starts to lose the long-range order at 400 °C (Figure S15). In consistency, the TGA shows that EM-L02 remains stable up to 400 °C, without significant weight loss, indicating the absence of significant water content (Figure S18). The observation from the in situ PXRD data fits with the onset of an exothermic peak in the DSC curve and a significant weight loss of 35.2% in the TGA curve with an onset at 402 °C, which closely matches the expected loss of 37.6%. The OSDA content was 31.7% according to CHN analysis (31.4% theoretically). It should be noted that the material contains a minor impurity of zeolite Beta, which remains stable after in situ PXRD measurements. The loss of long-range ordering of the *d6r* chains can be explained by the one-dimensional nature of EM-L02, which provides an increased degree of freedom, preventing the formation of a 3D framework material upon calcination.

### Scanning Transmission Electron Microscopy

EMM-75P, EMM-75, and EM-L01-TG were further studied by integrated differential phase contrast (iDPC) STEM images. Due to the preferred orientation, the samples were embedded in epoxy resin and sectioned using an ultramicrotome to a thickness of 50–70 nm.

The iDPC-STEM images obtained along the [100] direction of EMM-75P are consistent with the layered crystal structure as determined based on 3D ED data (see Figure 8a). Interestingly, the face perpendicular to [010] terminates with additional atoms to connect adjacent layers by supposedly forming two extra 5-rings. The crystal morphology suggests that growth along the [001] direction is hindered due to the weak interactions between layers to form the very thin layered structure. The crystal growth propagates fastest along the [100] direction, which coincides with the shortest unit cell axis as well as the π–π stacking direction of the OSDA.

**Figure 8:**
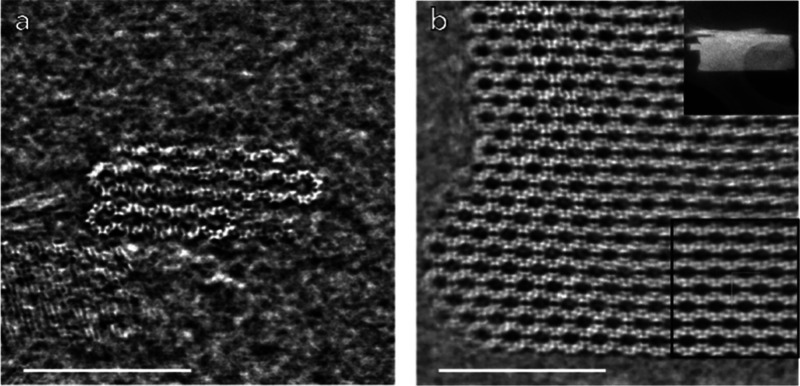
(a) The iDPC-STEM image of EMM-75P shows a view along the [100] direction of the structure, well in consistency with the EMM-75P crystal structure. (b) iDPC-STEM image of EMM-75 viewed along the [100] direction reveals a condensed crystal. Insets in (b) show ADF-STEM image of the crystal of EMM-75 (top) and the lattice-averaged map with *cmm* plane group symmetry imposed (bottom). The scale bars are 10 nm.

The calcined EMM-75 possesses larger plate-like crystal morphology, suggesting an aggregation of the smaller crystals of EMM-75P during calcination to form larger EMM-75 crystals, as shown in Figure 8b.

## Conclusions

Three new low-dimensional zeotype materials have been synthesized using similar cyclic benzimidazolium OSDAs. 3D ED data revealed their framework structures as well as the precise atom-by-atom location of the OSDA molecules in all three materials, the EMM-75P, EM-L01, and EM-L02. Based on the obtained structures, light was shed on the role of the OSDA in the formation of the structures of these low-dimensional zeolitic materials. The imidazolium group directs the growth of the zeolitic framework, where the π–π interactions between OSDA molecules promote the formation of straight channels, and the bulkier benzene group and the rigidity and limited mobility of the OSDAs prohibit the growth of three-dimensional framework structures.

Among the three materials, only EMM-75P topotactically condenses upon calcination to form a novel three-dimensional material, the new zeolite EMM-75, whereas EM-L01 partially condenses into the zeolite framework **STF**, and EM-L02 does not form a crystalline material upon calcination. A decisive factor for the success of the topotactic condensation appears to be the distance between terminal silanol groups, which is a consequence of the OSDA packing, and their interaction with the adjacent layers. Based on our study, the distance should ideally fall within the hydrogen bond interaction with an upper limit of ∼3.8 Å. In conclusion, the rigid and bulky nature of the OSDA molecules are key factors to form low-dimensional zeolitic materials, and the relative proximity of the terminal silanol groups is an important factor for a successful topotactic condensation.

This study provides a valuable understanding of the formation of low-dimensional zeotype materials synthesized by direct hydrothermal synthesis and the structure-directing role of the OSDAs. This will help to facilitate the synthesis of new low-dimensional zeotype materials, a viable route to obtain zeolite materials unfeasible to form by direct synthesis.

## Experimental Section

### Synthesis of Low-Dimensional Zeotypes

#### Synthesis of OSDAs

The synthesis of 2-ethyl-1,3-dimethylbenzimidazolium hydroxide and 2-ethyl-1,3-dimethyl-4,5,6,7-tetrahydrobenzimidazol-3-ium hydroxide is detailed in the Supporting Information.

#### Synthesis of EMM-75P

EMM-75P was synthesized hydrothermally using 2-ethyl-1,3-dimethylbenzimidazolium hydroxide (OSDA1­(OH)) as OSDA. A mixture of tetraethylorthosilicate (1.2 g, TEOS, >99 wt %) and Al­(OH)_3_ (0.035 g, Sigma, 54 wt % Al_2_O_3_) was hydrolyzed at room temperature in OSDA1 (OH solution (13.5 mL, 4 wt %) for about 2–3 h, and then HF (0.12 mL, 48 wt % solution) was added to the mixture. The resulting gel was aged at room temperature for a few days for evaporation of ethanol and water, yielding a synthesis mixture with the following molar composition: Si/Al = 15, OSDA1­(OH)/Si = 0.5, H_2_O/Si = 10, HF/Si = 0.5.

The resulting paste was homogenized by hand in a PTFE container and transferred to a 23 mL polytetrafluoroethylene (PTFE)-lined stainless-steel Parr autoclave. Crystallization was carried out at 135 °C under constant rotation (ca. 40 rpm) in the autoclave for 28 days in a convection oven. The product was isolated by filtration, rinsed with deionized water, and dried at 90 °C in a vented oven.

The as-synthesized material EMM-75P was then calcined to 580 °C for 8 h in air within a box furnace with a ramping rate of 3 °C/min to obtain EMM-75.

#### Synthesis of EM-L01

This material was synthesized under similar conditions as EMM-75P except that the Si/Al ratio was increased to 30.

#### Synthesis of EM-L02

The synthesis of EM-L02 was conducted with 2-ethyl-1,3-dimethyl-4,5,6,7-tetrahydrobenzimidazol-3-ium hydroxide (OSDA2­(OH)) as OSDA. Tetraethylorthosilicate (1.25 g, TEOS, >99 wt %) was hydrolyzed at room temperature in OSDA2­(OH) solution (3.8 mL, 15 wt %) for 2–3 h, and then HF (0.12 mL, 48 wt % solution) was added to the mixture. The resulting gel was aged at room temperature for a few days for ethanol and water evaporation, to produce a synthesis mixture having the composition: H_2_O/Si = 4, OSDA2­(OH)/Si = 0.5, HF/Si = 0.5.

The paste was homogenized manually in a PTFE container and transferred to a 23 mL PTFE-lined stainless steel Parr autoclave. The autoclave was kept at 160 °C with rotation (about 40 rpm) for 14 days in a convection oven. The final product was isolated by filtration, rinsed with deionized water, and dried at 90 °C in a vented drying oven.

### Characterization

#### Three-Dimensional Electron Diffraction Data Collection

3D ED data collection was performed using a JEOL JEM2100 LaB_6_ TEM operated at 200 kV, equipped with a Timepix hybrid pixel detector (Amsterdam Scientific Instruments), using the continuous rotation electron diffraction (cRED) method implemented in software Instamatic^39^ at room temperature. In the cRED method, the goniometer is continuously rotated in the microscope while frames are being collected.

The powders were crushed in a mortar, dispersed in ethanol (99%) and sonicated for 2 min and then transferred onto a Lacey carbon holey grid. A standard sample solution of Lu_3_Al_5_O_12_ ^40^ was added by drop casting of a dispersion to every grid as an internal standard to determine more accurate lattice parameters. The grid with the investigated sample and standard sample was then transferred to a single-tilt holder with a high-tilt retainer and loaded into the TEM.

#### Structure Solution and Refinement

X-ray Detector Software (XDS)^41^ was used to process the cRED data sets. The integrated and scaled intensities from XDS were used to solve and refine the structure using SHELXL-2018 ^42^ via OLEX2 ^43^ software.

#### Scanning Transmission Electron Microscopy

STEM images were collected using a FEI Themis Z aberration-corrected TEM operated at 300 kV. The images were collected with a semiconvergence angle of 16 mrad and dwell times 2, 2.5, and 10 μs for EMM-75P, EM-L01-TG, and EMM-75, respectively. Integrated differential phase contrast (iDPC) and annular dark-field (ADF) images were obtained simultaneously. The iDPC images were formed using a segmented annular detector with a 6–24 mrad collection angle. The ADF detector was set at a collection angle of 25–153 mrad. A high-pass filter was applied to the iDPC images to remove low-frequency noise. In order to enable imaging of the crystals along desired orientations, the powder was embedded into an epoxy resin and sectioned using an ultramicrotome to sections with a thickness of 50–70 nm.

#### Powder X-ray Diffraction

PXRD data were collected from EMM-75P, EM-L01, and EM-L02 using a Bruker D8 Discovery diffractometer with Cu Kα radiation (λCuKα1 = 1.5406 Å) in Bragg–Brentano geometry with motorized divergence slits equipped with a high-temperature cell. In situ PXRD data were collected between 5 and 50° (2θ) and a temperature range from 100 to 560 °C or 600 °C, the temperature was increased at a rate of 3 °C/min in air.

#### Thermal Gravimetric and Differential Scanning Calorimetry Analyses

TG was performed on a TA Instruments Discovery with a heating rate of 3 °C/min up to 800 °C in a 20 mL/min flow of air. DSC analysis was performed on Netzsch DSC 214 Polyma with a heating rate of 3 °C/min from room temperature to 560 °C with a purge air atmosphere of 60 mL/min.

#### Solid-State Nuclear Magnetic Resonance

All the solid-state NMR measurements were done at room temperature and with ^1^H decoupling during data acquisition. Solid-state ^13^C, ^19^F, and ^27^Al MAS NMR spectra were recorded at 14.1 T using a Bruker Avance NEO 600 MHz standard-bore spectrometer (where the corresponding Larmor frequencies of ^13^C, ^19^F, and ^27^Al are 150.9, 564.5, and 156.4 MHz, respectively). The samples were loaded in either 3.2 or 4 mm-outer diameter (o.d.) MAS rotors and spun at the magic angle at a rate of 12–20 kHz.

The ^13^C CPMAS NMR spectra were acquired using a pulse delay of 2 s and a contact time of 2 ms. ^13^C chemical shifts were referenced using a secondary standard, solid adamantane with a chemical shift of 38.5 ppm.

The ^19^F MAS NMR spectra were acquired using a pulse delay of 5 s. ^19^F chemical shifts were referenced using a secondary standard, neat liquid HFB (hexafluorobenzene) with a chemical shift of −165 ppm.

The ^27^Al MAS NMR spectra were acquired using a pulse delay of 0.5 s. ^27^Al chemical shifts were referenced using a secondary standard, solid Al­(NO_3_)_3_ with a chemical shift of −3 ppm.

The ^29^Si solid-state NMR spectra were recorded at 9.4 T on a Varian InfinityPlus 400 MHz wide-bore spectrometer (where the corresponding Larmor frequency of ^29^Si is 79.5 MHz). The samples were loaded in 7.5 mm o.d. MAS rotors and spun at the magic angle at a rate of 4 kHz. The ^29^Si MAS spectra were obtained using a pulse delay of 30 s. The chemical shifts were referenced using a secondary standard, Q_8_M_8_ (octakis­(trimethylsiloxy)­silsesquioxane) with a chemical shift (δ_Si_-CH_3_) of 12.40 ppm.

#### Elemental Analysis

The elemental analysis that probed the C, N, and H content was obtained using ThermoFisher Flash 2000 CHNS/O.

#### Scanning Electron Microscopy

The SEM images of EMM-75P, EM-L01, and EM-L02 were collected on a JEOL JSM IT-800 with a Shottky-type field-emission gun. Images were collected with secondary electron and backscattered electron detectors.

## Supplementary Material



## Notes

Experimental section, synthesis details of the zeotype materials, OSDAs, characterization details, and crystallographic information (PDF)

## Abbreviations

3D ED: three-dimensional electron diffraction
CBU: compositing building units
OSDA: organic structure-directing agent
SCXRD: single-crystal X-ray diffraction
cRED: continuous rotation electron diffraction
PXRD: powder X-ray diffraction
TGA: thermogravimetric analysis
DSC: differential scanning calorimetry
STEM: scanning transmission electron microscopy
NMR: nuclear magnetic resonance
MAS NMR: magic-angle spinning nuclear magnetic resonance
CPMAS NMR: cross-polarization magic-angle spinning nuclear magnetic resonance
XDS: X-ray Detector Software
