# Supplementary material for: Low-Dimensional Zeotypes Templated by Stacked Cyclic Benzimidazolium Revealed by Electron Crystallography
Source: J Am Chem Soc. 2026 Feb 5;148(6):6686–94. doi: 10.1021/jacs.5c22569 (PMC12921870; doi:10.1021/jacs.5c22569)
Supplement: Supplementary file 1 [file ja5c22569_si_001.pdf]

Supporting Information

Low-Dimensional Zeotypes Templated By Stacked Cyclic Benzimidazolium Revealed By Electron Crystallography

Evgeniia Ikonnikova[a], Jung Cho[a], Xiaodong Zou[a], Andre Sutrisno[b], Allen W. Burton[b], Trong Pham*[b], Tom Willhammar*[a]

[a] Department of Chemistry, Stockholm University, Stockholm SE-106 91, Sweden

[b] ExxonMobil Technology & Engineering Company, 1545 Route 22 East, Annandale, New Jersey 08801, United States

Contents

Synthesis of Organic Structure Directing Agents (OSDAs) 3

2-ethyl-1,3-dimethylbenzimidazolium (OSDA1): 3

2-ethyl-1,3-dimethyl-4,5,6,7-tetrahydrobenzimidazol-3-ium (OSDA2): 3

Micropore Analysis 4

Elemental analysis 4

Structure Solution and Refinement 4

Figures and Tables 5

Figure S1. (a) $^{1}H/^{13}C$ CP MAS NMR and (b) ^{19}F MAS NMR spectra of as-made EMM-75P sample. Spinning side bands are marked by asterisks. 6

Figure S2. (a) $^{1}H/^{13}C$ CP MAS NMR and (b) ^{19}F MAS NMR spectra of as-made EM-L01 sample. Spinning side bands are marked by asterisks. 7

Figure S3. (a) $^{1}H/^{13}C$ CP MAS NMR and (b) ^{19}F MAS NMR spectra of as-made EM-L02 sample. Spinning side bands are marked by asterisks. 8

Figure S4. Powder X-ray Diffraction patterns of (a) EMM-75P, (b) EMM-75, (c) EM-L01, (d) calcined EM-L01, (e) EM-L02, (f) calcined EM-L02 ($\lambda CuK\alpha 1$ = 1.5406 Å) 10

Figure S5. ^{27}Al MAS NMR spectra of (a) EMM-75P and (b) EM-L01 11

Figure S6. ^{29}Si NMR spectra of (a) EMM-75P and (b) EMM-75 11

Figure S7. ^{29}Si NMR spectra of (a) as-made and (b) calcined EM-L01 12

Figure S8. ^{29}Si NMR spectra of as-made EM-L02 12

Figure S9. Reconstruction 3D reciprocal lattice of EMM-75P 13

Table S1. Continuous rotation electron diffraction data collection and structure refinement details of EMM-75P. 14

Figure S10. Reconstruction 3D reciprocal lattice from cRED data of (a) EM-L01 and (b) EM-L01-TG. 14

Table S2. Continuous rotation electron diffraction data collection and structure refinement details of EM-L01. 15

Figure S11. Reconstruction 3D reciprocal lattice from cRED data of EM-L02. 16

Table S3. Continuous rotation electron diffraction data collection and structure refinement details of EM-L02. 16

Figure S12. Reconstruction 3D reciprocal lattice from cRED data of EMM-75. 16

Table S4. Continuous rotation electron diffraction data collection and structure refinement details of EMM-75. 17

Figure S13. Natural tilings of (a-b) EMM-75P and EMM-75. Composite building units shown for (c-d) EM-L01, (e) EM-L02.. 17

Figure S14. Results from in situ PXRD thermal investigation with a step of 3°C/min on EM-L01 (λCuKα1 = 1.5406 Å). 18

Figure S15. Results from in situ PXRD thermal investigation with a step of 3°C/min on EM-L02 (λCuKα1 = 1.5406 Å). 18

Figure S16. Thermal analysis of EMM-75P 19

Figure S17. Thermal analysis of layered EM-L01 20

Figure S18. Thermal analysis of EM-L02 21

Figure S19. iDPC-STEM images of EM-L01-TG.. 21

Figure S20. Pawley fit of (a) EMM-75P, (b) EM-L01, (c) EM-L02 and (d) EMM-75 22

Table S5. Unit cell parameters statistic from continuous rotation electron diffraction data versus powder X-ray diffraction data 22

References 23

Synthesis of Organic Structure Directing Agents (OSDAs)

2-ethyl-1,3-dimethylbenzimidazolium (OSDA1):

In a round-bottom flask, 2-ethyl-benzimidazole (15.0 g) was dissolved in 170 mL of acetonitrile (CH_3CN). Potassium carbonate (21.0 g) was then added to the solution, and, iodomethane (58.3 g) was added dropwise. The reaction mixture was heated to 60°C overnight. Upon cooling to room temperature, potassium carbonate was filtered out from the solution, and the filtrate was removed under reduced pressure. Chloroform was added to the flask to precipitate remaining potassium salts, and the resulting mixture was filtered again. The filtrate was rotavaporated in chloroform to obtain pure 2-ethyl-1,3-dimethyl-1H-benzo[d]imidazole-3-ium iodide.

The iodide salt was then ion-exchanged to its hydroxide form with resin Amberlite® IRN78 OH, with a weight ratio of 1: 3.5: 5 (iodide: resin: water). The exchange was performed overnight at room temperature. The solution was then recovered by filtration of the resin and washing with deionized water. The aqueous fractions were then combined and concentrated under rotoevaporation at around 60°C. The hydroxide concentration was determined by titration with a standard 0.1N HCl solution.

2-ethyl-1,3-dimethyl-4,5,6,7-tetrahydrobenzimidazol-3-ium (OSDA2):

2-Ethyl-4,5,6,7-tetrahydro-1*H*-benzimidazole:

The target OSDA2 was synthesized in three steps. Initially, to a flask of 320 ml of glacial acetic acid, 2-Ethylbenzimidazole (40.0 g) was added under stirring. Then palladium (10 wt% on carbon) (15.0 g) was added to the solution, and the mixture was treated with hydrogen at a temperature of 120°C under a pressure of 80 bar for 24 hours in a 600 ml Parr reactor. The solution was filtered over celite and washed with glacial acetic acid. (Caution: throughout handling of the Pd/C, the catalyst should be kept moist with the solvent to avoid charring or fires.). The solvent was evaporated under vacuum and the pH was adjusted to 9-10 by adding sodium hydroxide solution. Later, the precipitate was collected by filtration, washed with water, dissolved in chloroform and then extracted with saturated sodium chloride solution. The organic phase was dried over sodium sulfate, filtered and concentrated under vacuum.

In the second step, to a 250 mL round-bottom flask equipped with a magnetic stir bar, 2-ethyl-4,5,6,7-tetrahydro-1H-benzimidazole (14 g), iodomethane (70 g) and potassium carbonate (27 g) were added. The suspension was subsequently refluxed for 18 hours, then cooled to room temperature. After the filtration through a Buchner funnel, the filtrate solution was concentrated by rotoevaporation. Dichloromethane was added to the concentrated solution to precipitate any remaining potassium salts. Afterwards, the filtrate in dichloromethane was concentrated by rotoevaporation and the recovered product was dried under vacuum to obtain 2-ethyl-1,3-dimethyl-4,5,6,7-tetrahydrobenzimidazol-3-ium iodide.

In the final step, the iodide form of the OSDA2 was ion-exchanged to hydroxide form with resin Amberlite® IRN78 OH with a ratio of 1 : 3.5: 5 (iodide: resin: water). The exchange was performed at room temperature overnight.

Micropore Analysis

Nitrogen physisorption isotherms were measured at 77 K using a Micromeritics 3Flex device. Prior to the measurements, each sample was activated at a temperature of 623 K for 6 h in vacuum. The overall Brunauer-Emmett-Teller (BET) surface area (S_{BET}) of the materials was determined by the BET method using nitrogen adsorption-desorption data obtained at 77K[1]. The external surface area (S_{ext}) of the material was obtained from the t-plot method. The micropore surface area (S_{micro}) of the material was calculated by subtracting the S_{ext} from the overall S_{BET}. The micropore volume (V_{micro}) and total pore volume (V_{tot}) of the materials can be determined by the t-plot method[2].

Based on the measurements of the EMM-75 the S_{micro} was 174 m^2/g, S_{ext} was 45 m^2/g, V_{tot} was 0.23 cm^3/g and V_{micro} was 0.074 c cm^3/g.

Elemental analysis

The silicon to aluminum (Si/Al) ratios of zeolite and zeolitic materials were determined by X-ray fluorescence (XRF) analysis operated on a S2 PUMA Bruker instrument. The measured Si/Al ratios were 13 for EMM-75 and 23.5 for EM-L01, respectively.

Carbon, Hydrogen and Nitrogen (CHN) analysis was performed by dynamic flash combustion method (following ASTM D5291, method D) using a ThermoFisher Flash 2000 CHNS/O Elemental Analyzer. Approximately 2-3 mg of each sample was packed into a tin capsule, transferred to an autosampler drum, and purged with a continuous flow of helium and oxygen (UHP grade, 99.995% pure) gases into a combustion/oxidation furnace of 950 C. The mixture is subsequently passed through a gas chromatography (GC) column and quantitatively measured using a thermal conductivity detector (TCD).

Calibration and quality control (QC) checks were done using 2,5-bis (5-tert-butyl-benzoxazol-2-yl) thiophene (BBOT) with known values of 72.53% C, 6.09% H and 6.51% N. Based on the measurements, the OSDA content in EMM-75P, EM-L01 and EM-L02 is 22.3%, 24.6% and 31.7%, accordingly.

Structure Solution and Refinement

The 3D reciprocal space was reconstructed using REDp. To determine the unit cell of the sample, a peak search was initially conducted by adjusting the peak searching parameters. This was followed by peak merging, where the intensity of each reflection was integrated. The space group was determined based on reflection conditions from 2D cuts.

The data processing involved several steps: spot finding, indexing, integration, and scaling. Initially, the merged datasets were independently indexed, and the individual ASCII files were then scaled together using XSCALE. The unit cell parameters for the merged datasets were calculated by using those of the individual datasets. Unreliable data were identified based on the final statistics of I/sigma and CC1/2. The structure solution was subsequently performed using SHELXT. In the cases of EMM-75 and EM-L01, all atoms of the framework and the OSDA were placed ab initio, while for EM-L02, manual identification of part of the OSDA was required.

The refinement was carried out using SHELXL within the OLEX2 software. The refinement process employed a least-squares algorithm to improve the model. A free variable (FVAR) was used as a refinable constraint to adjust the occupancy of the OSDA, treating all OSDA atoms as a single component. The bond length of T-O and tetrahedral bond angle O-T-O were optimized using the DFIX and DANG constraints. Oxygen atoms of water molecules were identified based on the peak maxima in the difference positive electrostatic potential map and filtered according to their σ-level. The hydrogen atoms of terminated T atoms T-O-H and of OSDA atoms were placed by the command Add H in OLEX2. The refinement was considered complete when the shift between the model and the experimental data reached 0.0.

Figures and Tables

Solid-state ^{13}C Cross-Polarization Magic Angle Spinning Nuclear Magnetic Resonance (CP MAS NMR) spectroscopy was employed extensively to probe the structural integrity and spatial confinement of OSDAs occluded within the microporous frameworks of zeolitic materials. The chemical shift assignments corresponding to the carbon environments of the encapsulated organic cations, as presented in Figures S1a, S2a, and S3a, confirm the preservation of the molecular structure of the OSDAs post-synthesis. Notably, the broader linewidths observed in Figures S1a and S2a, relative to those in Figure S3a, suggest a more rigid local environment and restricted molecular mobility. This is attributed to π–π stacking interactions among aromatic moieties within the confined voids of the EMM-75P and EM-L01 frameworks, which impose conformational constraints on the occluded species.

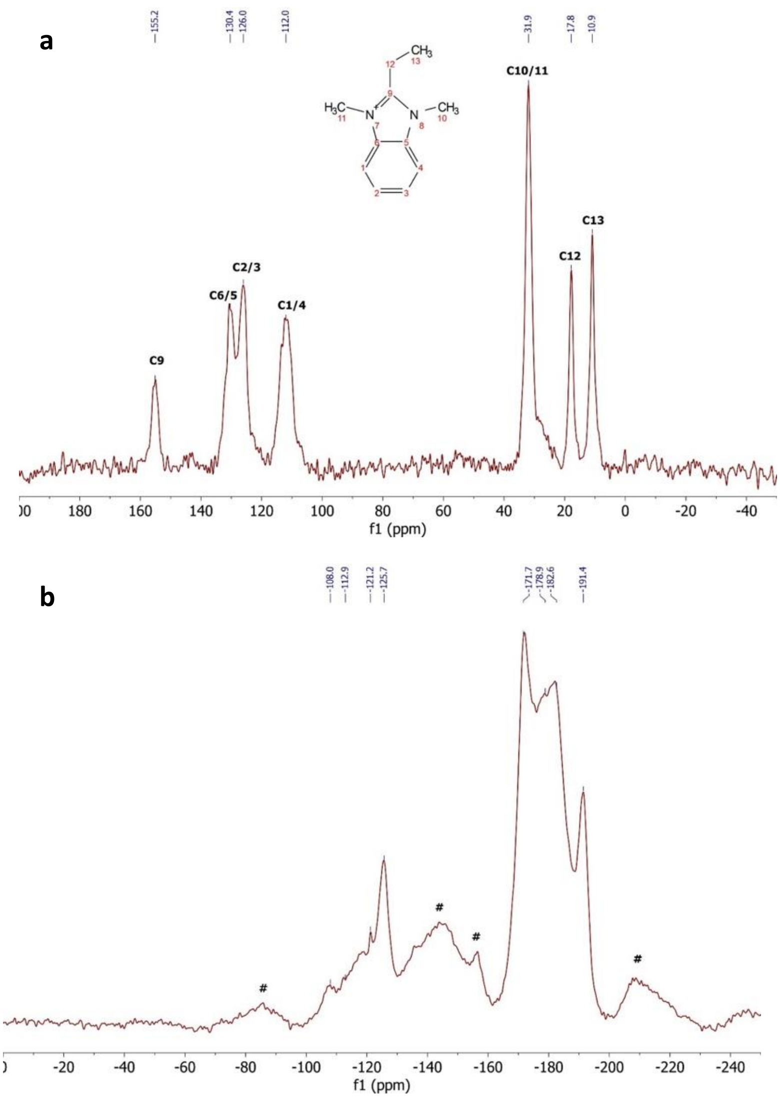

Figure S1. (a) $^{1}H/^{13}C$ CP MAS NMR and (b) ^{19}F MAS NMR spectra of as-made EMM-75P sample. Spinning side bands are marked by hash symbols.

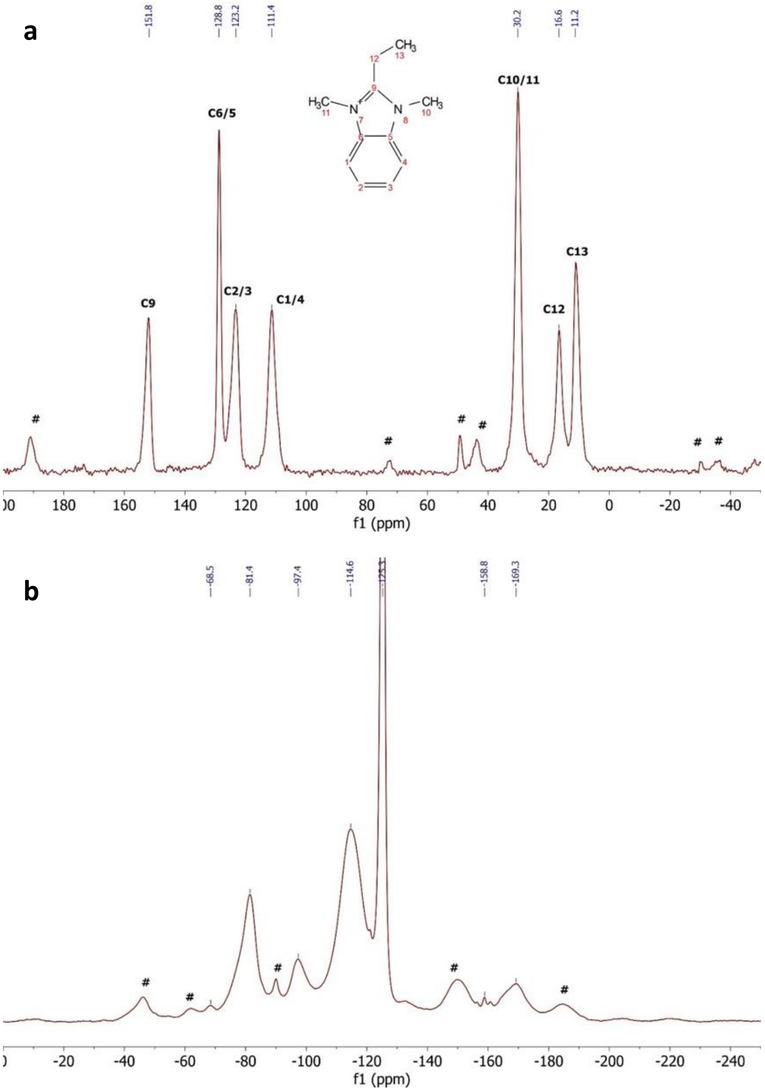

Figure S2. (a) $^{1}H/^{13}C$ CP MAS NMR and (b) ^{19}F MAS NMR spectra of as-made EM-L01 sample. Spinning side bands are marked by hash symbols.

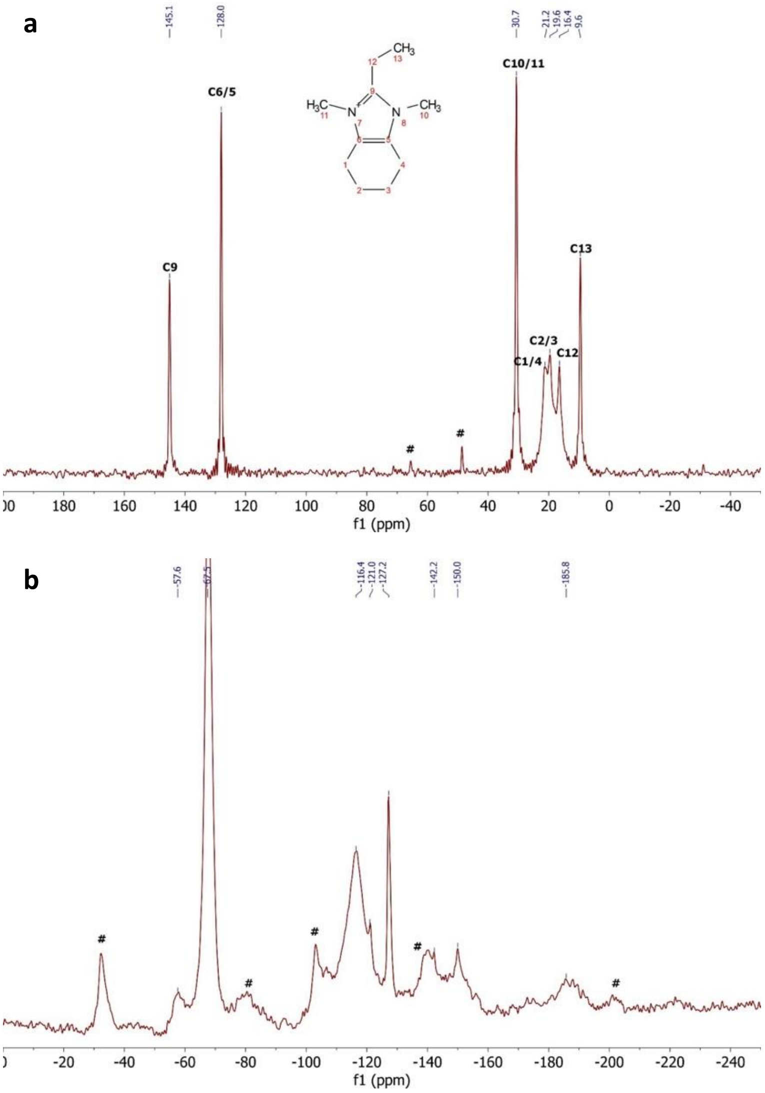

Figure S3. (a) $^{1}H/^{13}C$ CP MAS NMR and (b) ^{19}F MAS NMR spectra of as-made EM-L02 sample. Spinning side bands are marked by hash symbols.

In Figures S1b, S2b, and S3b present the ^{19}F MAS NMR spectra of the as-synthesized EMM-75P, EM-L01, and EM-L02 zeolitic materials. The spectra exhibit both broad and sharp resonances, indicative of multiple fluorine environments within the framework. These signals are attributed to fluoride species interacting with various framework and extra-framework components. Specifically, the observed chemical shifts in the range of approximately −80 to −130 ppm are consistent with fluoride anions associated with OSDAs, protonated species (HF), and silicon-fluoride complexes such as SiF_6^{2-} and SiF_4^{-}, as reported in the literature[3–6]. The coexistence of sharp and broad features suggests a distribution of dynamic and static fluoride environments, likely reflecting differences in local coordination and mobility within the zeotypes pores.

In the ^{19}F MAS NMR spectrum of the EMM-75P (Figure S1b), multiple resonances were observed in the range of −160 to −190 ppm, which are attributed to the presence of aluminum-fluoride coordination species. These signals are consistent with the formation of Al–F complexes, such as $[AlF_6]^{3-}$ or partially coordinated AlF_x species, which are known to resonate in this spectral window due to their highly shielded fluorine environments and strong Al–F bonding interactions[6,7]. As expected, EMM-75P with high aluminum content (Si/Al~13) lacks any detectable ^{19}F resonances in the region between −35 and −80 ppm corresponding to F^- ions occluded in small cavities of zeolites.

The ^{19}F chemical shift at -80 ppm of EM-L01 sample (Figure S2b) is consistent with the presence of the five-coordinate $[SiO_{4/2}F]^-$ unit in the fused $[4^1 5^2 6^2]$ cages, as reported by Morris *et al*[8]. The resonance at −67 ppm is due to F in the *d6r* cages of the EM-L02 material (Figure S3b), similar to what was observed in *d6r* s of the CHA structure[9], and has a structure-directing role in the absence of alkali cations[10–12].

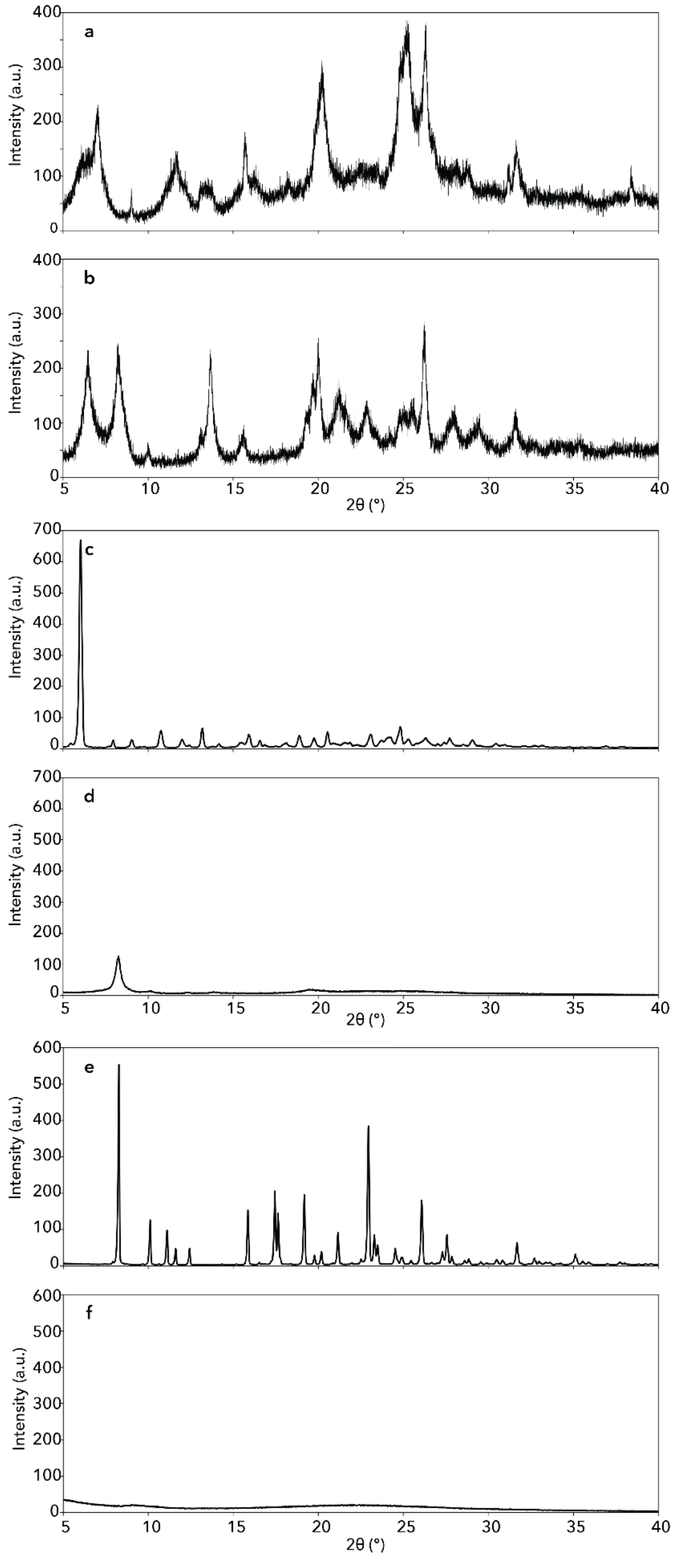

Figure S4. Powder X-ray Diffraction patterns of (a) EMM-75P, (b) EMM-75, (c) EM-L01, (d) calcined EM-L01, (e) EM-L02, (f) calcined EM-L02 ($\lambda CuK\alpha 1 = 1.5406$ Å).

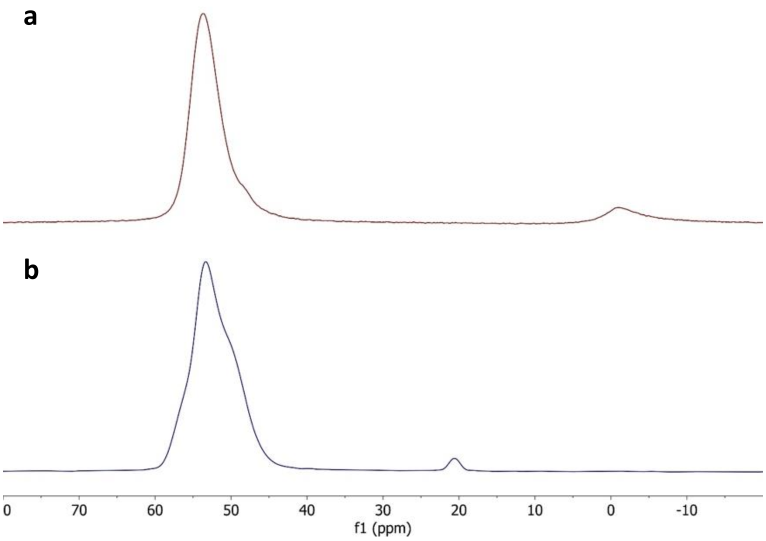

Figure S5. ^{27}Al MAS NMR spectra of (a) EMM-75P and (b) EM-L01.

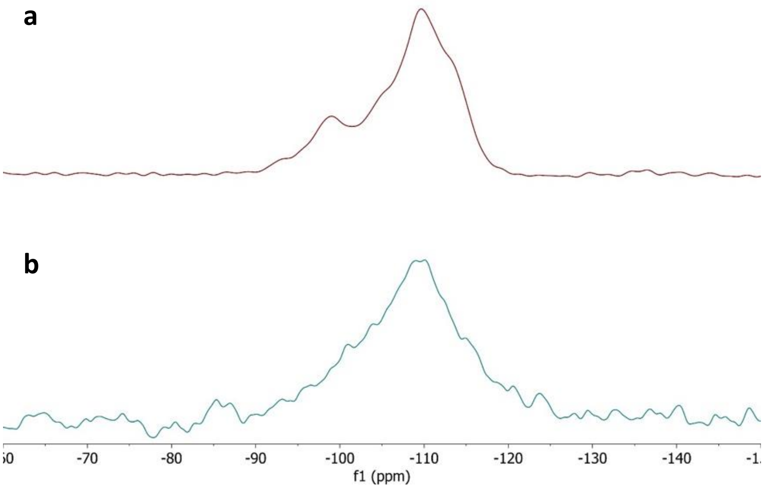

Figure S6. ^{29}Si NMR spectra of (a) EMM-75P and (b) EMM-75.

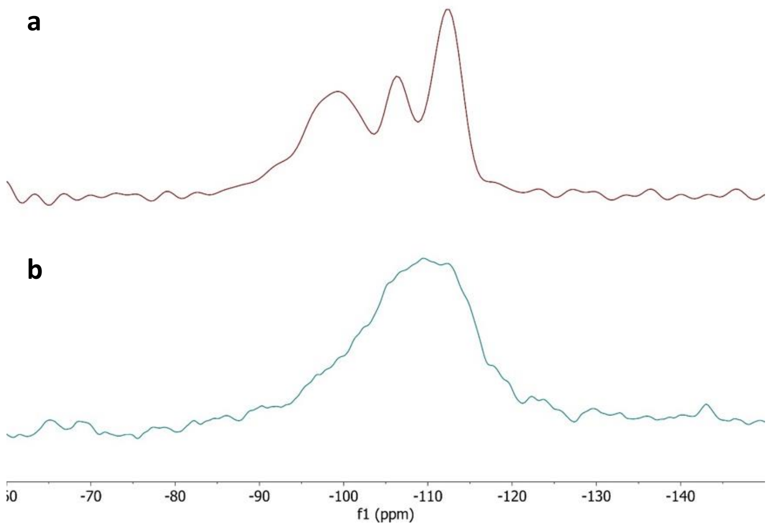

Figure S7. ^{29}Si NMR spectra of (a) as-made and (b) calcined EM-L01.

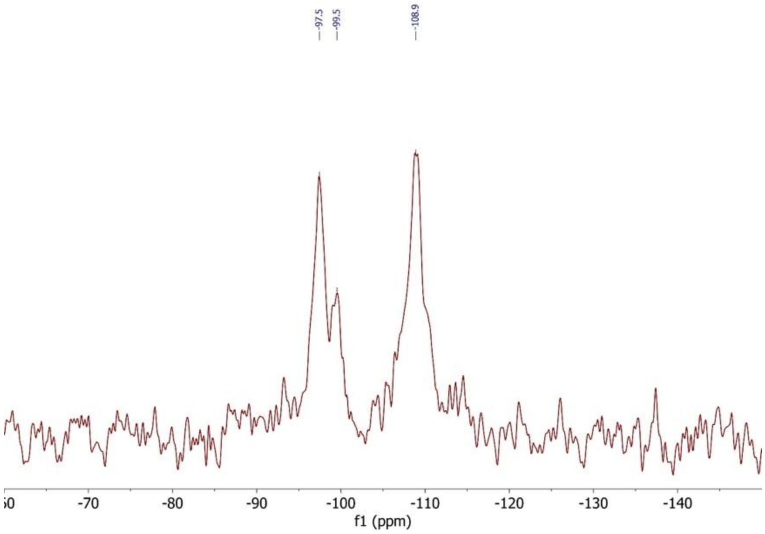

Figure S8. ^{29}Si NMR spectra of as-made EM-L02.

The ^{27}Al MAS NMR spectra (Figure S5a) reveal that EMM-75P predominantly contains tetrahedrally coordinated framework aluminum species (Al^{IV}) centered at ~55 ppm, accounting for approximately 90% of the total aluminum signal. A minor broad resonance (~10%) near 0 ppm is attributed to hexacoordinated extra-framework aluminum species (Al^{VI}). The ^{29}Si NMR spectra (Figure S6a) of EMM-75P show peaks centered at –110 ppm and –115 ppm, which are attributed to the silicate framework units (≡Si–O–Si≡), specifically Q^4(0Al) sites. Peaks at –105 ppm and –100 ppm correspond to Brønsted acid sites (≡Si–OH–Al≡, Q^4(1Al)) and silanol groups (≡Si–OH, Q^3). The reduced intensity of the -100 ppm peak after calcination is attributed to reduction of Q^3 silanol groups, see Figure S6b.

In contrast, the ^{27}Al MAS NMR spectrum of EM-L01 exhibits a strong signal corresponding to framework Al^{IV}, with no detectable resonance at 0 ppm, indicating the absence of significant extra-framework Al species (Figure S5b). A sharp, low-intensity peak (~2%) at ~20 ppm is assigned to hexa-coordinated aluminum (Al^{VI}), possibly originating from trace Al_2O_3 impurities deposited on the zeolite surface during or post-synthesis[13]. The ^{29}Si NMR spectra shows the resonances centered at –112 ppm and –107 ppm are attributed to fully condensed Q^4 Si(0Al) environments, indicative of a well-connected silicate framework in the EM-L01 material, see Figure S7a. Similar to observations for EMM-75, the reduction in intensity following calcination was observed in EM-L01 around -100 ppm attributed to Q^3 reduction, which might also overlap with contributions from Brønsted acid sites (≡Si–OH–Al≡, Q^4(1Al)) (Figure S7b).

The ^{29}Si MAS NMR spectrum of EM-L02 (Figure S8) indicates a high degree of framework connectivity by exhibiting a resonance at –109 ppm, characteristic of fully condensed silica species (Q^4, Si(0Al)). Also, notable signals centered at –98 and –100 ppm are attributed to Q^3 species, corresponding to silanol groups (≡Si–OH).

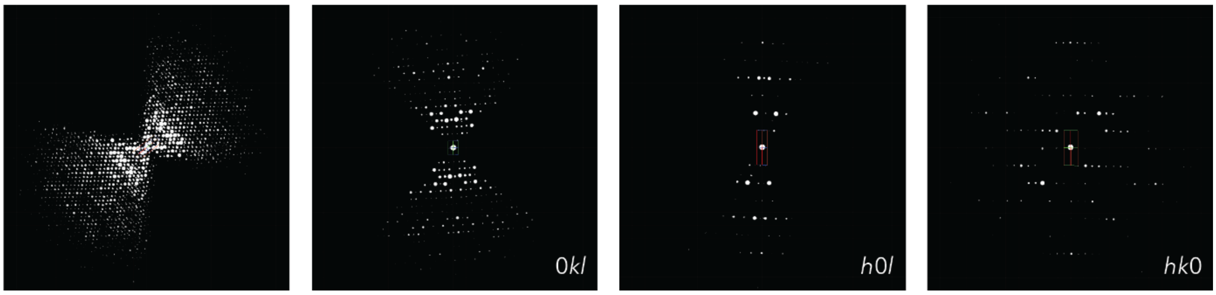

Figure S9. Reconstruction 3D reciprocal lattice of EMM-75P from cRED data using REDp software. Three 2-dimensional slices, 0*kl*, *h*0*l*, and *hk*0 extracted from the reconstructed reciprocal lattice.

Table S1. Continuous rotation electron diffraction data collection and structure refinement details of EMM-75P. Unit cells for merged data were calculated using three individual datasets.

	**Dataset 1**	**Dataset 2**	**Dataset 3**	**Merged data**
Rotation range, °	65.4	100.7	77.1	
Tilt step per frame, °		0.23		
Exposure time/frame, s		0.5		
No. cRED frames	281	433	332	
Crystal system			monoclinic	
Resolution, Å	0.8	0.9	0.9	0.9
Space group (no.)	$P2_1/n$ (14)	$P2_1/n$ (14)	$P2_1/n$ (14)	$P2_1/n$ (14)
a, Å	7.32(8)	7.32(13)	7.33(3)	7.33(1)
b, Å	17.87(2)	17.90(6)	17.83(10)	17.73(1)
c, Å	25.20(10)	25.17(9)	25.39(19)	25.17(1)
β, °	92.72(12)	94.01(3)	93.88(10)	92.54(3)
Wavelength, Å			0.02508	
Observed reflections	6311	7296	6160	19195
Unique reflections	3237	3220	2677	3350
Completeness, %	48.2	68.1	56.4	71.2
No.restraints/ No.parameters				61/195
Goodness-of-fit on F^2				1.069
Final R indexes [I>=2σ (I)], %				R_1 = 0.1854, wR_2 = 0.4607
Final R indexes [all data], %				R_1 = 0.2317, wR_2 = 0.5049

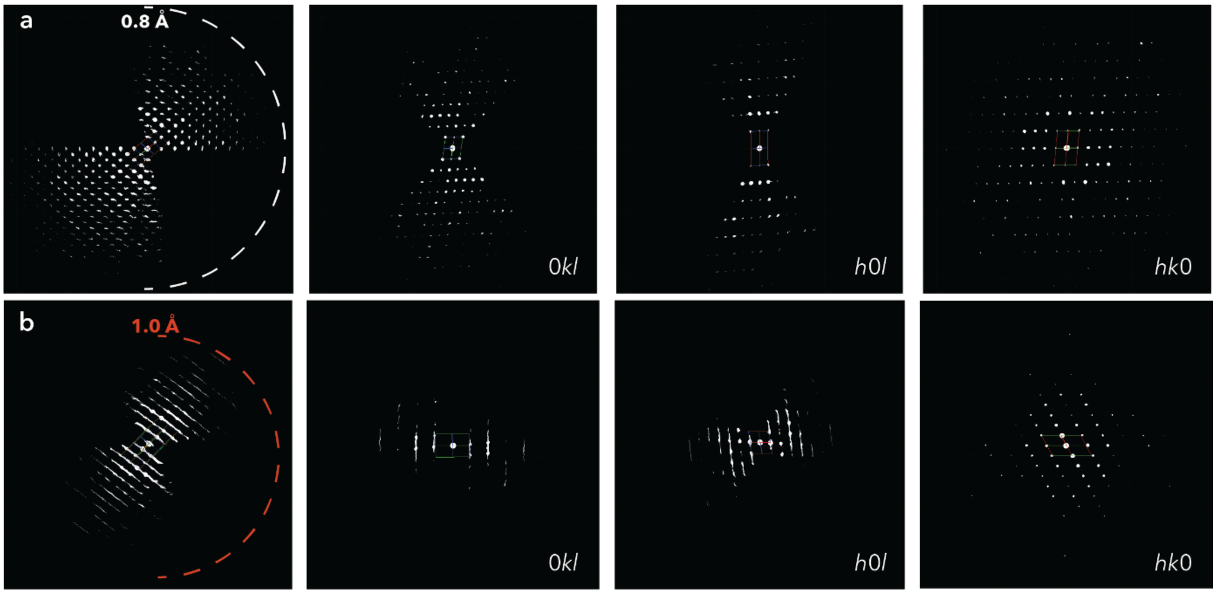

Figure S10. Reconstruction 3D reciprocal lattice from cRED data of (a) EM-L01 and (b) EM-L01-TG. Three 2-dimensional slices, 0*kl*, *h*0*l*, and *hk*0 extracted from the reconstructed reciprocal lattice. The semicircles indicate the resolution of the individual datasets.

Table S2. Continuous rotation electron diffraction data collection and structure refinement details of EM-L01**.** Unit cells for merged data were calculated using six individual datasets.

	**Dataset 1**	**Dataset 2**	**Dataset 3**	**Dataset 4**	**Dataset 5**	**Dataset 6**	**Merged data**
Rotation range, °	108.9	110.0	104.0	79.3	95.6	104.8	
Tilt step per frame, °	0.23						
Exposure time/frame, s	0.5						
No. cRED frames	422	426	403	307	370	406	
Crystal system	triclinic						
Resolution, Å	0.84	0.8	0.8	0.8	0.8	0.8	0.8
Space group (no.)	*P*-1 (2)	*P*-1 (2)	*P*-1 (2)	*P*-1 (2)	*P*-1 (2)		*P*-1 (2)
a, Å	7.39(2)	7.42(1)	7.33(2)	7.45(2)	7.44(3)	7.42(2)	7.41(2)
b, Å	11.38(1)	11.38(9)	11.44(2)	11.46(1)	11.44(1)	11.46(1)	11.43(2)
c, Å	14.47(3)	14.94(3)	14.68(3)	14.74(3)	14.83(4)	14.70(3)	14.73(6)
α, °	79.25(4)	79.52(16)	79.37(9)	79.99(8)	79.36(11)	79.32(4)	79.38(9)
β, °	88.06(4)	87.12(4)	86.11(17)	88.79(12)	87.68(24)	88.34(11)	87.64(25)
γ, °	83.17(9)	83.49(5)	83.37(19)	83.34(9)	83.21(8)	83.12(7)	83.29(6)
Wavelength, Å	0.02508						
Observed reflections	4671	5197	4799	3448	4329	4891	27275
Unique reflections	2697	2945	2735	2036	2437	2742	3832
Completeness, %	55.4	58.5	56.1	40.7	48.4	55.4	77.4
No.restraints/ No.parameters							34/159
Goodness-of-fit on F^2							1.039
Final R indexes [I>=2σ (I)], %							R_1 = 0.2051, wR_2 = 0.4993
Final R indexes [all data], %							R_1 = 0.2583, wR_2 = 0.5655

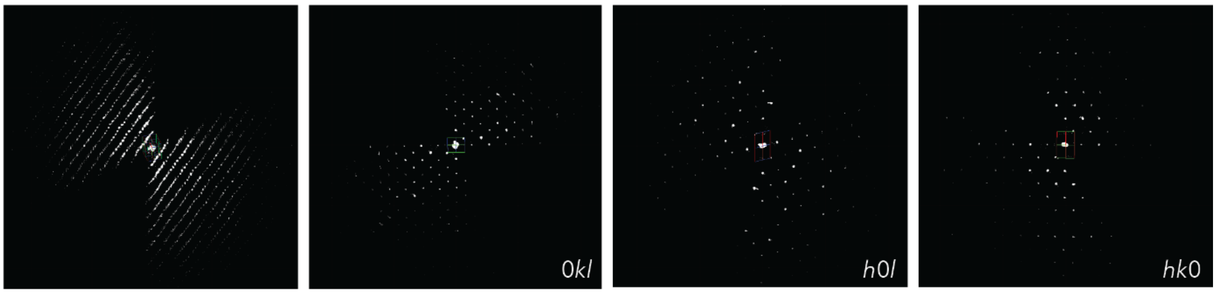

Figure S11. Reconstruction 3D reciprocal lattice from cRED data of EM-L02. Three 2-dimensional slices, 0*kl*, *h*0*l*, and *hk*0 extracted from the reconstructed reciprocal lattice.

Table S3. Continuous rotation electron diffraction data collection and structure refinement details of EM-L02.

Rotation range, °	119.3
Tilt step per frame, °	0.23
Exposure time/frame, s	0.5
No. cRED frames	463
Crystal system	monoclinic
Resolution, Å	0.79
Space group (no.)	*C*2/*m* (12)
a, Å	17.09(1)
b, Å	14.51(1)
c, Å	9.25(1)
α, °	90
β, °	108.80(3)
γ, °	90
Volume, $Å^3$	2171.4(8)
Completeness, %	91.8
Wavelength, Å	0.0251
Observed reflections	4616
Unique reflections	2134
No.restraints/ No.parameters	26/84
Goodness-of-fit on F^2	1.147
Final R indexes [I>=2σ (I)]	R_1 = 0.1879, wR_2 = 0.4825
Final R indexes [all data]	R_1 = 0.2107, wR_2 = 0.5076

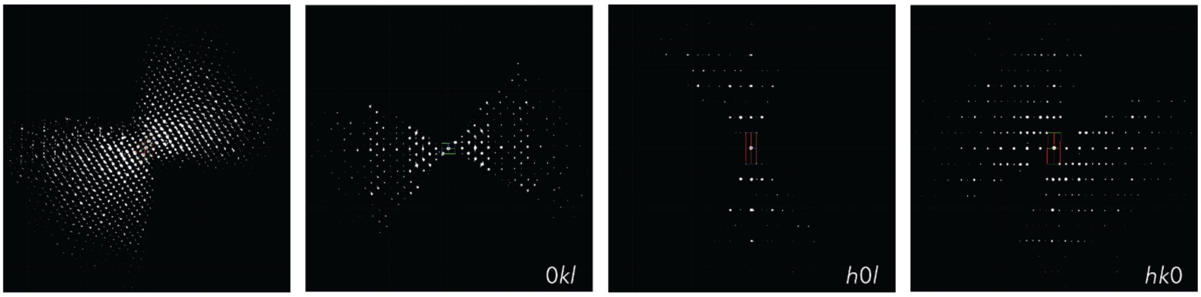

Figure S12. Reconstruction 3D reciprocal lattice from cRED data of EMM-75. Three 2-dimensional slices, 0*kl*, *h*0*l*, and *hk*0 extracted from the reconstructed reciprocal lattice.

Table S4. Continuous rotation electron diffraction data collection and structure refinement details of EMM-75.

Tilt range, °	94.2
Tilt step per frame, °	0.23
Exposure time/frame, s	0.5
No. cRED frames	365
Crystal system	orthorhombic
Resolution, Å	0.81
Space group (no.)	*Pnnm* (58)
a, Å	7.36(10)
b, Å	17.77(32)
c, Å	21.57(72)
Volume, $Å^3$	2824.4(11)
Completeness, %	75.3
Wavelength, Å	0.0251
Observed reflections	8718
Unique reflections	2187
No.restraints/ No.parameters	14/74
Goodness-of-fit on F2	1.195
Final R indexes [I>=2σ (I)]	R_1 = 0.1934, wR_2 = 0.4736
Final R indexes [all data]	R_1 = 0.2313, wR_2 = 0.5077

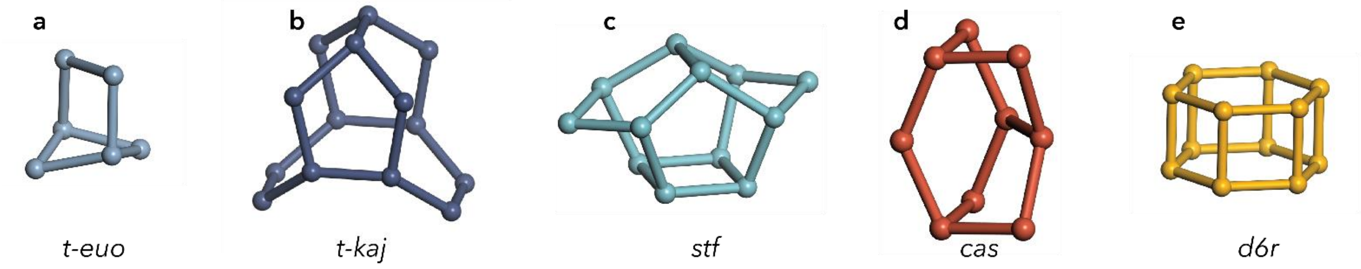

Figure S13. Natural tilings of (a-b) EMM-75P and EMM-75. Composite building units shown for (c-d) EM-L01, (e) EM-L02.

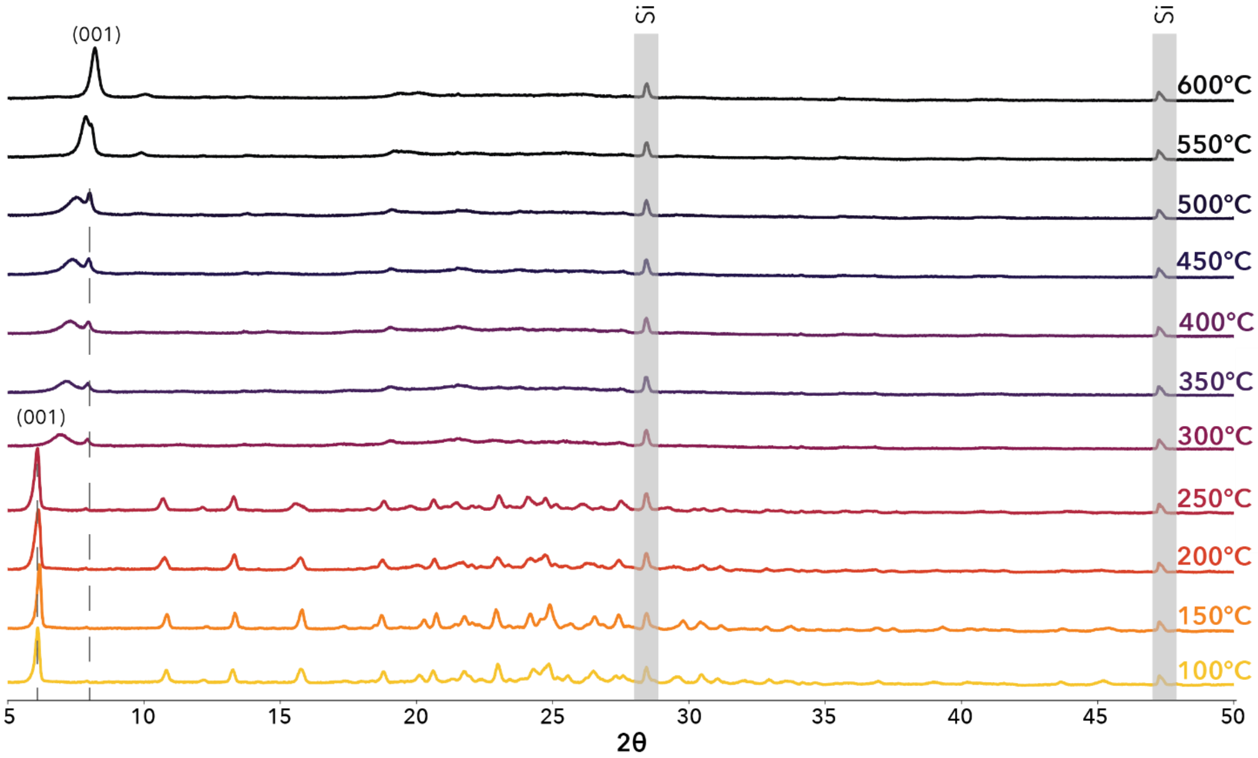

Figure S14. Results from in situ PXRD thermal investigation with a step of 3°C/min on EM-L01 (λCuKα1 = 1.5406 Å). Si powder was used as an internal standard.

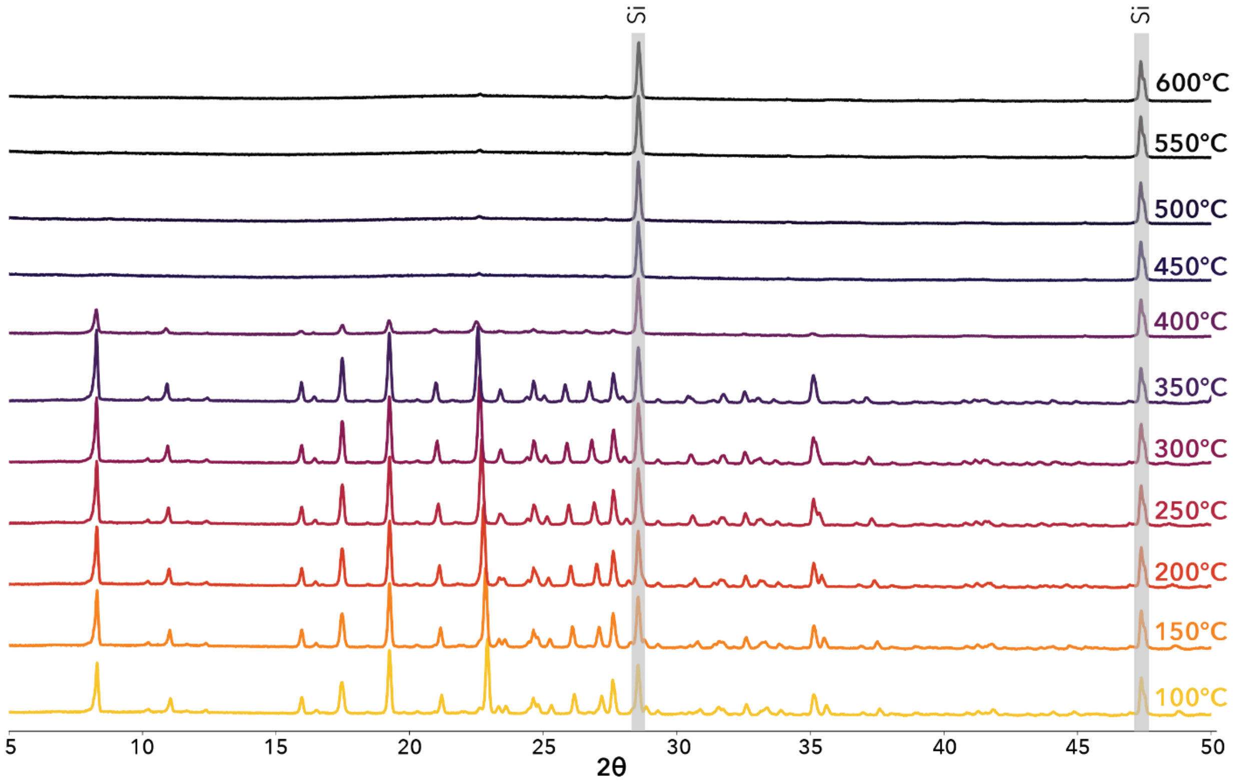

Figure S15. Results from in situ PXRD thermal investigation with a step of 3°C/min on EM-L02 (λCuKα1 = 1.5406 Å). Si powder was used as an internal standard.

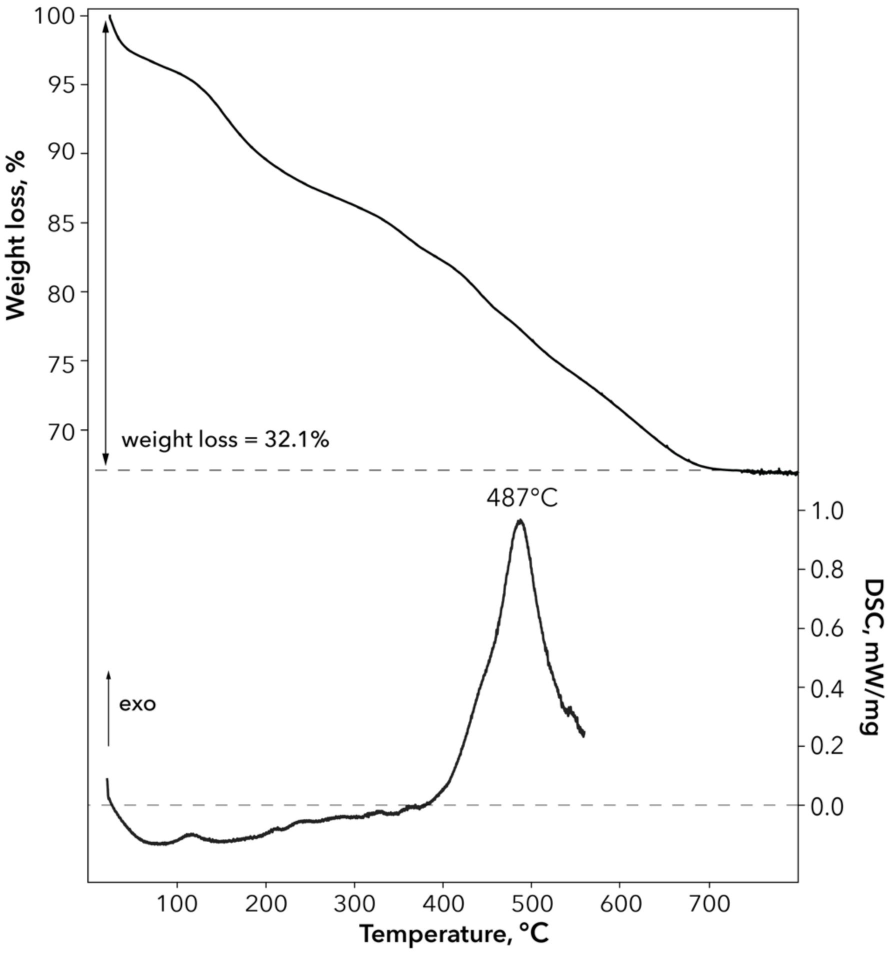

Figure S16. Thermal analysis of EMM-75P (a) TGA under air, (b) DSC under air.

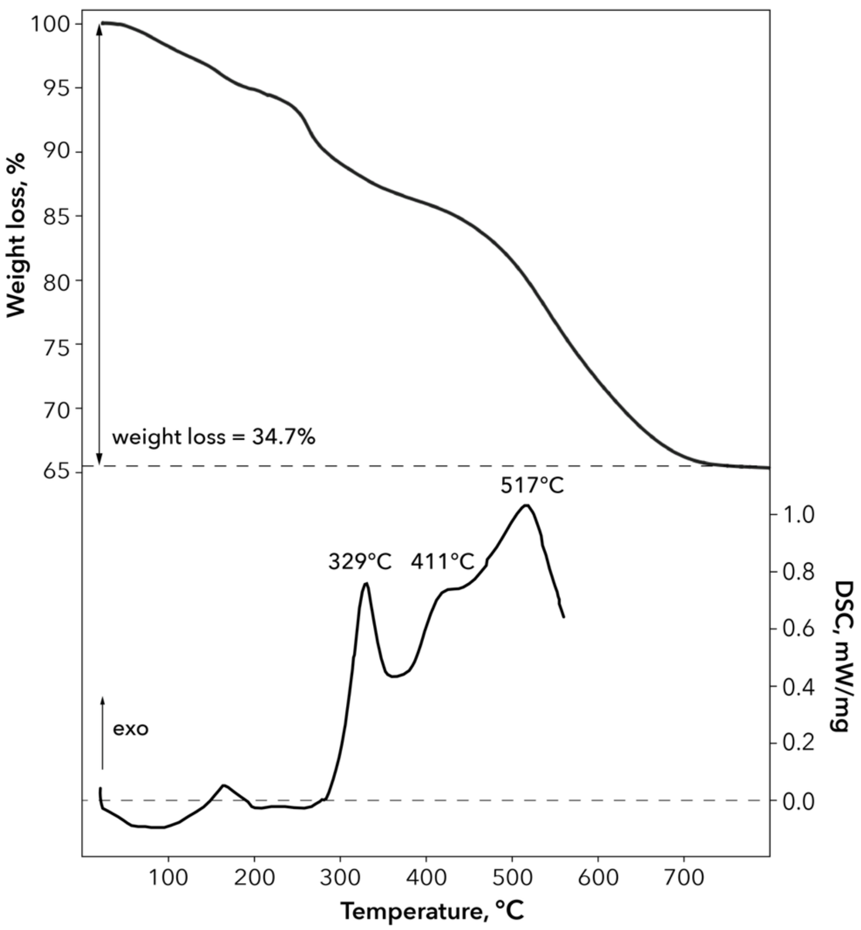

Figure S17. Thermal analysis of layered EM-L01 (a) TGA under air, (b) DSC under air.

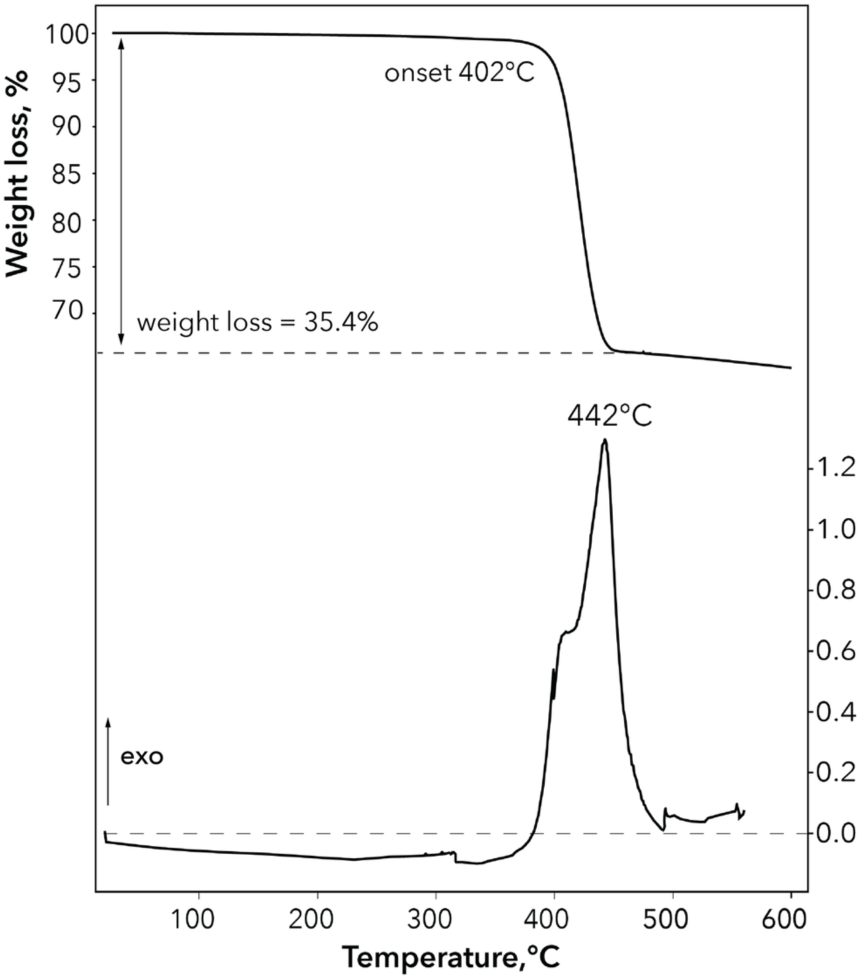

Figure S18. Thermal analysis of EM-L02 (a) TGA under air, (b) DSC under air.

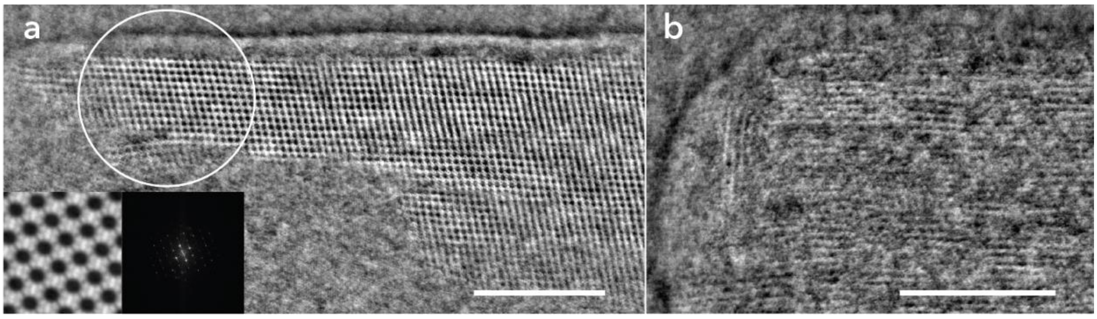

Figure S19. iDPC-STEM images of EM-L01-TG along [100]. (a) A crystal condensed into the STF topology, whereas (b) from the same batch shows a highly disordered structure. Insets in (a) show (left) the lattice-averaged map with $p2$ plane group symmetry imposed and (right) FFT from the selected region. The scale bars are 20 nm.

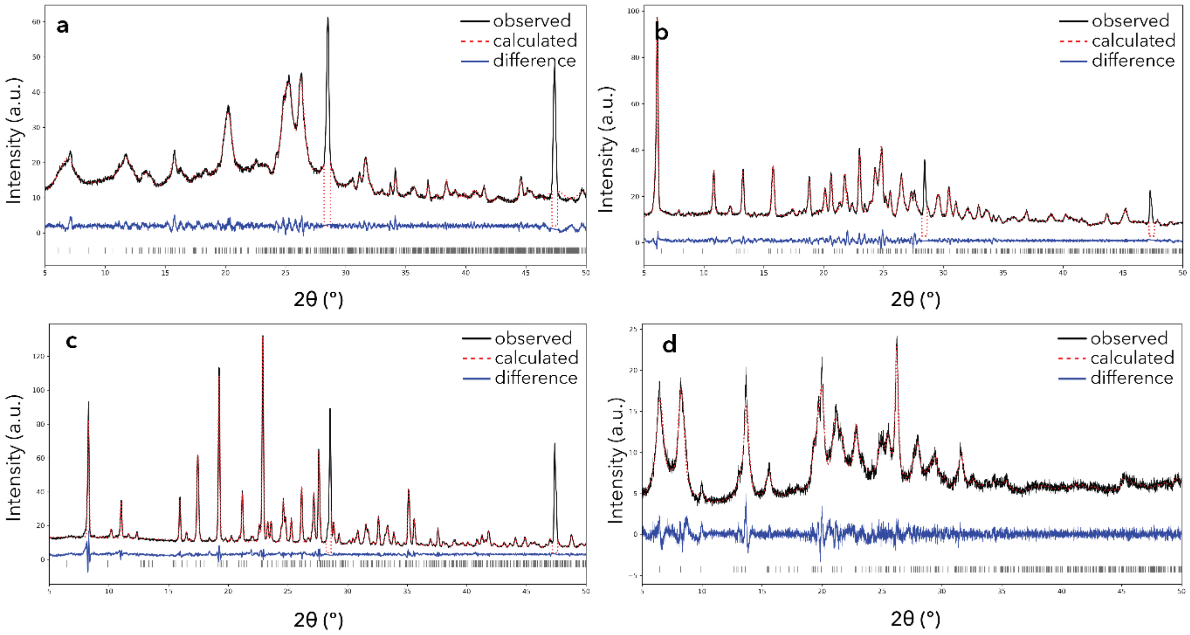

Figure S20. Pawley fit of (a) EMM-75P, (b) EM-L01, (c) EM-L02 and (d) EMM-75**.** Excluded peaks from the Pawley fits are corresponding to Si, which was used as an internal standard**.** (λCuKα1 = 1.5406 Å).

Table S5. Unit cell parameters statistic from continuous rotation electron diffraction data versus powder X-ray diffraction data

Sample/unit cell parameters	**EMM-75P**		**EMM-75**		**EM-L01**		**EM-L02**	
	3D ED	PXRD	3D ED	PXRD	3D ED	PXRD	3D ED	PXRD
a, Å	7.33(1)	7.5(2)	7.36(10)	7.365(3)	7.41(2)	7.456(3)	17.09(3)	17.086(1)
b, Å	17.73(1)	17.6(6)	17.77(32)	17.840(10)	11.43(2)	11.434(3)	14.51(3)	14.506(6)
c, Å	25.17(1)	25.1(4)	21.57(72)	21.424(7)	14.73(6)	14.683(8)	9.25(2)	9.247(7)
α, °	90	90	90	90	79.38(9)	79.31(2)	90	90
β, °	92.54(3)	96(2)	90	90	87.65(25)	86.98(2)	108.80(3)	108.809(5)
γ, °	90	90	90	90	83.30(6)	83.28(3)	90	90

References

(1) Brunauer, S.; Emmett, P. H.; Teller, E. Adsorption of Gases in Multimolecular Layers. *J. Am. Chem. Soc.* **1938**, *60* (2), 309–319. https://doi.org/10.1021/ja01269a023.

(2) Lippens, B. Studies on Pore Systems in Catalysts V. The t Method. *Journal of Catalysis* **1965**, *4* (3), 319–323. https://doi.org/10.1016/0021-9517(65)90307-6.

(3) Miller, J. M. Fluorine-19 Magic-Angle Spinning NMR. *Prog. Nucl. Magn. Reson. Spectrosc.* **1996**, *28* (3–4), 255–281. https://doi.org/10.1016/0079-6565(95)01024-6.

(4) Lin, Z. S.; Chen, D.; Nie, H.-Y.; Wong, Y. T. A.; Huang, Y. Investigations of the Formation of Zeolite ZSM-39 (MTN). *Can. J. Chem.* **2019**, *97* (12), 840–847. https://doi.org/10.1139/cjc-2019-0076.

(5) Bae, J.; Hong, S. B. Choline-Mediated Synthesis of Zeolite ERS-7 *via* an Excess Fluoride Approach. *Chem. Commun.* **2018**, *54* (78), 10997–11000. https://doi.org/10.1039/C8CC06106H.

(6) Delmotte, L.; Soulard, M.; Guth, F.; Seive, A.; Lopez, A.; Guth, J. L. 19F MAS n.m.r. Studies of Crystalline Microporous Solids Synthesized in the Fluoride Medium. *Zeolites* **1990**, *10* (8), 778–783. https://doi.org/10.1016/0144-2449(90)90061-U.

(7) Kao, H.-M.; Chen, Y.-C. ^{27}Al and ^{19}F Solid-State NMR Studies of Zeolite H-β Dealuminated with Ammonium Hexafluorosilicate. *J. Phys. Chem. B* **2003**, *107* (15), 3367–3375. https://doi.org/10.1021/jp021680q.

(8) Fyfe, C. A.; Brouwer, D. H.; Lewis, A. R.; Villaescusa, L. A.; Morris, R. E. Combined Solid State NMR and X-Ray Diffraction Investigation of the Local Structure of the Five-Coordinate Silicon in Fluoride-Containing As-Synthesized STF Zeolite. *J. Am. Chem. Soc.* **2002**, *124* (26), 7770–7778. https://doi.org/10.1021/ja012558s.

(9) Villaescusa, L. A.; Bull, I.; Wheatley, P. S.; Lightfoot, P.; Morris, R. E. The Location of Fluoride and Organic Guests in 'as-Made' Pure Silica Zeolites FER and CHA. *J. Mater. Chem.* **2003**, *13* (8), 1978–1982. https://doi.org/10.1039/B300728F.

(10) Cao, G.; Strohmaier, K. G.; Li, H.; Guram, A. S.; Saxton, R. J.; Muraoka, M. T.; Yoder, J. C.; Yaccatu, K. Aei-Type Zeolite, Its Synthesis and Its Use in the Conversion of Oxygenates to Olefins. WO2005063624A1, July 14, 2005. https://patents.google.com/patent/WO2005063624A1/en.

(11) Díaz-Cabañas, M.-J.; Barrett, P. A. Synthesis and Structure of Pure SiO2 Chabazite: The SiO2 Polymorph with the Lowest Framework Density. *Chem. Commun.* **1998**, No. 17, 1881–1882. https://doi.org/10.1039/a804800b.

(12) Wragg, D. S.; Morris, R.; Burton, A. W.; Zones, S. I.; Ong, K.; Lee, G. The Synthesis and Structure of SSZ-73: An All-Silica Zeolite with an Unusual Framework Topology. *Chem. Mater.* **2007**, *19* (16), 3924–3932. https://doi.org/10.1021/cm0705284.

(13) Slade, R. C. T. 27AI Nuclear Magnetic Resonance Spectroscopy Investigation of Thermal Transformation Sequences of Alumina Hydrates. *J. Mater. Chem.* **1991**, *1*. https://doi.org/10.1039/JM9910100563.
